# Identification of human nephron progenitors capable of generation of kidney structures and functional repair of chronic renal disease

**DOI:** 10.1002/emmm.201201584

**Published:** 2013-09-02

**Authors:** Orit Harari-Steinberg, Sally Metsuyanim, Dorit Omer, Yehudit Gnatek, Rotem Gershon, Sara Pri-Chen, Derya D Ozdemir, Yaniv Lerenthal, Tzahi Noiman, Herzel Ben-Hur, Zvi Vaknin, David F Schneider, Bruce J Aronow, Ronald S Goldstein, Peter Hohenstein, Benjamin Dekel

**Affiliations:** 1 The Pediatric Stem Cell Research Institute, Edmond and Lily Safra Children's Hospital, Sheba Center for Regenerative Medicine, Sheba Medical CenterRamat-Gan, Israel; 2 Mina and Everard Goodman Faculty of Life Sciences, Bar-IlanUniversityRamat-Gan, Israel; 3 Sackler School of Medicine, Tel Aviv UniversityTel Aviv, Israel; 4 The Roslin Institute, University of Edinburgh, Easter Bush CampusMidlothian, UK; 5 Cancer Research Center, Sheba Medical CenterRamat-Gan, Israel; 6 L.E.M. Laboratory of Early DetectionNes Ziona, Israel; 7 Department of Obstet and Gynecology, Assaf HarofehTzrifin, Israel; 8 Division of Molecular and Developmental Biology, Department of Pediatrics, University of Cincinnati, Childrens Hospital Medical CenterCincinnati, OH, USA; 9 Division of Pediatric Nephrology, Edmond& Lily Safra Children's Hospital, Sheba Medical CenterRamat-Gan, Israel

**Keywords:** development, kidney stem cells, progenitor cells, regeneration, stem cells

## Abstract

Identification of tissue-specific renal stem/progenitor cells with nephrogenic potential is a critical step in developing cell-based therapies for renal disease. In the human kidney, stem/progenitor cells are induced into the nephrogenic pathway to form nephrons until the 34 week of gestation, and no equivalent cell types can be traced in the adult kidney. Human nephron progenitor cells (hNPCs) have yet to be isolated. Here we show that growth of human foetal kidneys in serum-free defined conditions and prospective isolation of NCAM1^+^ cells selects for nephron lineage that includes the SIX2-positive cap mesenchyme cells identifying a mitotically active population with *in vitro* clonogenic and stem/progenitor properties. After transplantation in the chick embryo, these cells—but not differentiated counterparts—efficiently formed various nephron tubule types. hNPCs engrafted and integrated in diseased murine kidneys and treatment of renal failure in the 5/6 nephrectomy kidney injury model had beneficial effects on renal function halting disease progression. These findings constitute the first definition of an intrinsic nephron precursor population, with major potential for cell-based therapeutic strategies and modelling of kidney disease.

## INTRODUCTION

Nearly 26 million Americans, one in every nine, harbour kidney disease (Trivedi, 2010). Despite recent medical advances, the treatment options for patients with renal failure are limited. The alternatives available to patients who succumb to terminal renal disease are either supportive treatment in the form of dialysis or whole organ replacement by kidney transplantation. Dialysis is associated with long-term morbidity, mortality and poor quality of life. The shortage of donor organs and the long wait time on the recipient list hamper renal transplantation (Daar, 2006). The number of patients with terminal renal disease has increased, and the treatment costs for these patients now exceed the cumulative costs of treating cancer patients (Trivedi, 2010). Against this background of the growing number of patients with kidney disease and the limited treatment options, alternative treatments are clearly in need.

Various types of stem cells may be applicable as a platform for cell therapy for renal disease (Pleniceanu et al, 2010; Harari-Steinberg et al, 2011). Nevertheless, it seems that: (a) bone marrow and blood stem cells do not generate nephron cell types (Duffield et al, 2005; Krause & Cantley, 2005; Dekel et al, 2006b) and (b) the existence of an adult kidney epithelial stem cell with wide nephrogenic potential is still tenuous. Hence, isolation of tissue stem/progenitor cells from foetal kidneys is an attractive option for replenishment of nephron cells (Pleniceanu et al, 2010; Harari-Steinberg et al, 2011). The mammalian kidney is formed via reciprocally inductive interactions between two mesoderm precursor tissues, the metanephric mesenchyme (MM) and the ureteric bud (UB) (Pleniceanu et al, 2010). In response to UB signals, induced MM cells acquire an epithelial phenotype (mesenchymal to epithelial transition; MET) to generate committed nephron progenitor populations and sequentially form the pretubular aggregate, renal vesicle and C- and S-shaped bodies that expand to give rise to the mature nephron (Pleniceanu et al, 2010). Recent lineage tracing experiments of cell populations in transgenic mouse models have established that the transcription factor Six2 signifies a multipotent progenitor cell in the MM that condensates to form cap mesenchyme (CM) around the UB and is capable of self-renewing and of differentiating towards different types of nephron epithelia (Boyle et al, 2008; Kobayashi et al, 2008).

Nevertheless, only a few studies have utilized human foetal kidney (hFK) as starting material for regenerative purposes, and most employ tissue transplantation rather than derivation of specific human cell types suitable for *in vitro* manipulation and cell therapy (Dekel et al, 1997, 2002, 2003; Hammerman, 2000). Progenitor cell types in the MM have been previously isolated from embryonic mouse kidneys by means of a supply of the nephrogenic inducer, Wnt4 (Osafune et al, 2006), or by growing cells as nephrospheres, which, although robustly propagated *in vitro*, failed to display renal epithelial/nephrogenic capacity (Lusis et al, 2010).

With the intention of prospectively isolating progenitor populations from hFK by flow cytometry, we carried out global gene analysis (Dekel et al, 2006a) followed by protein expression studies (Metsuyanim et al, 2009) to pinpoint phenotypic surface markers that would replace intra-cellular renal transcription factors not relevant for sorting from human tissue, among them the neural cell adhesion molecule 1 (NCAM1). In the current study, by utilizing clonal analysis, defined serum-free culture conditions, and cell sorting with NCAM1, we were able, for the first time, to isolate a progenitor population from mid-gestation hFKs strongly committed to nephron formation and displaying beneficial effects in a mouse model of chronic renal failure.

## RESULTS

### NCAM1^+^ cells are enriched for embryonic renal stem cell markers

**Figure 1:**
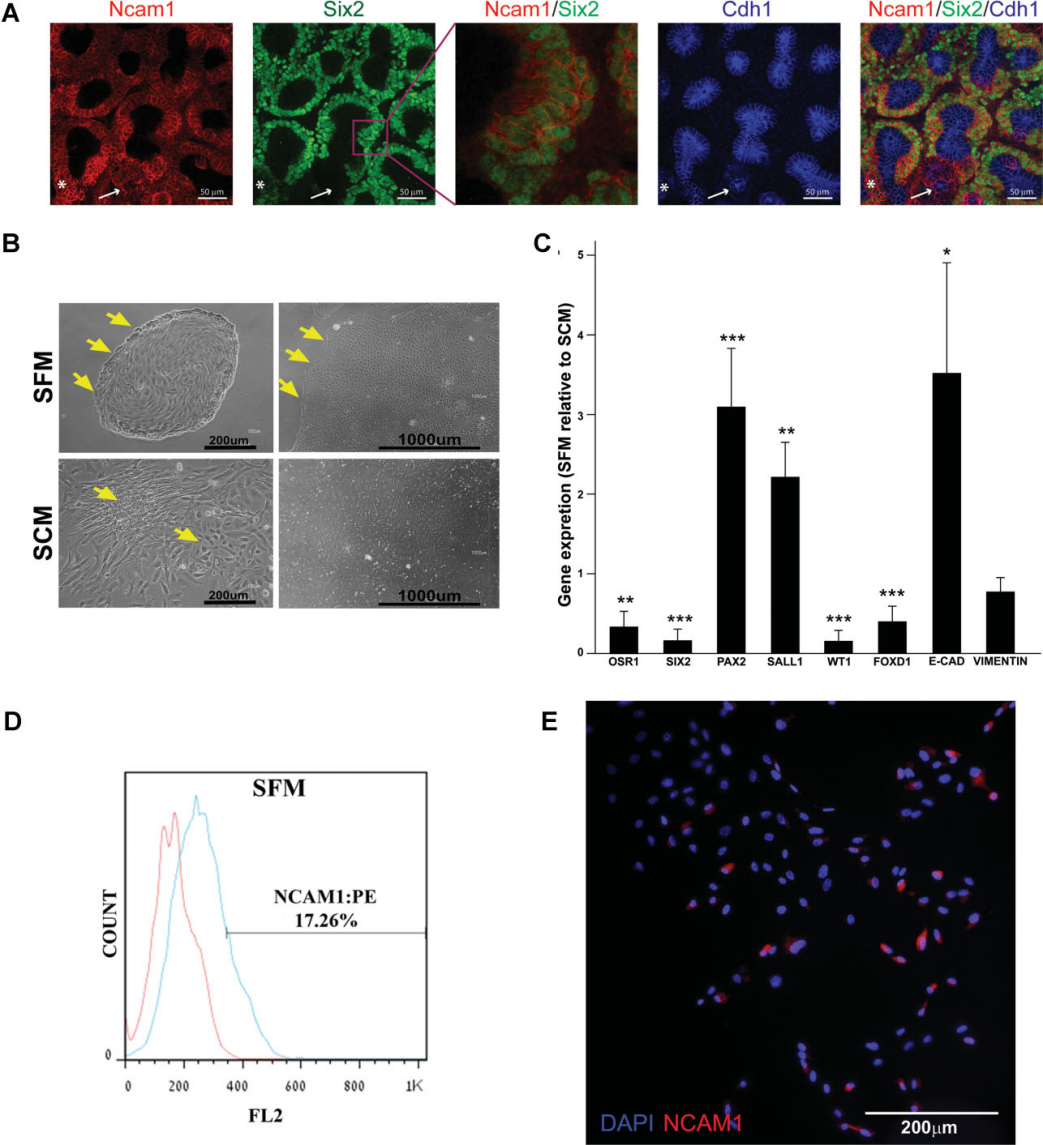
NCAM1 expression in mouse embryonic kidney organ and hFK serum-free cultures Mouse embryonic kidney organ culture stained for Ncam1, Six2 and E-cad as indicated. An enlargement of the Ncam/Six2 signal is shown to emphasize the nuclear localization of Six2. White arrow illustrates the absence of Six2 signal in E-cad positive cells. An occurrence of Six2/E-cad positive cells is indicated with the asterisk. Images were obtained using Nikon A1R confocal microscope with and processed in ImageJ/Fiji software.Morphology of hFK cells cultured in SFM or SCM after 3 days (passage 0 Day 3—left panels) and towards confluence (14 days in SFM or 7 days in SCM—right panels). Distinct borders appear in SFM cultures (arrows) whereas cells with different morphology (arrows) are observed in SCM culture. Cells were observed using a Nikon Digital Sight camera attached to a Nikon Eclipse TS100 microscope.qRT-PCR analysis of gene expression in hFK cells cultured in SFM (three independent replicates). *hPRT1* was used as endogenous control and SCM cells were used as the calibrator sample for RQ calculation (therefore = 1). Data were analysed using SDS 3.2 software.Representative FACS analysis of NCAM1 expression in hFK cells cultured in SFM at passage1. Data is presented in a histogram graph showing NCAM1 staining in blue and the isotype controls staining (negative control) in red.Immunofluorescence staining of NCAM1 (red) in total hFK cells cultured in SFM. Nuclei stained with Dapi (blue). Images were obtained using Olympus DP72 camera attached to Olympus BX51 fluorescence microscope and processed via cellSens standard software.

There has been considerable effort to identify cell surface markers to prospectively isolate stem cell populations from the kidney. We have previously put forward NCAM1 as a candidate marker for this (Dekel et al, 2006a; Metsuyanim et al, 2009). In fact, repeated verification by microarray analysis comparing human foetal and adult kidneys revealed *NCAM1* as the most differentially expressed cell surface marker (>23-fold). Work in transgenic mouse models has identified Six2 in the CM as self-renewing nephron stem/progenitor cells (Kobayashi et al, 2008). To compare expression patterns in the developing kidney we co-stained organ cultures of embryonic mouse kidneys with antibodies for Ncam1, Six2 and Cdh1/E-cadherin (Fig 1A). As previously described in hFK sections (Metsuyanim et al, 2009), Ncam1 in mouse organ culture was found expressed in the CM, as well as the earliest differentiated structures (renal vesicle, comma and S-shaped bodies). Six2 expression was solely found in the CM. Expression of Cdh1 as marker for epithelial cells was found to be almost mutually exclusive with Six2 expression, confirming the pre-MET expression of Six2, although occasionally we found co-expression of Six2 and Cdh1 as cells were going through the MET. It is not clear if these cells still express new Six2 protein, or whether they simply have not lost the existing protein yet. Therefore, Ncam1 expression was found in pre- and post-MET stages.

We queried a genomic data set from the GUDMAP database based on cell-type-specific gene expression profiles derived from a series of GFP-transgenic mouse lines and FACS separated lineages, each representing a specific developmental compartment regulated temporally and spatially (Harding et al, 2011). In the mixed kidney sample ST 1.0 dataset, Ncam1 was clearly and strikingly elevated in P0–P3 CM, corresponding to the time of post-natal nephrogenic burst observed in the mouse (Ncam1 is strongly expressed in P1 CM, data not shown). To identify other genes whose expression was co-ordinately regulated with that of Ncam1, we used Pearson correlation across these normalized gene expression profiles and identified a group of genes that exhibit essentially identical activation in P0-P3 CM and become somewhat inhibited at P4, consistent with the cessation of nephrogenic burst activity (Supporting Information Fig S1). The Ncam1 expression module, highly active in the peripartum nephrogenic burst phase CM, remarkably disclosed an overlap among neurogenic and nephrogenic programs and highlighted key players in the nephrogenic differentiation process (Supporting Information Fig S2). Interestingly, many of these genes are targeted by miRs-200b and 204, which regulate epithelial–mesenchymal transitions (Mongroo & Rustgi, 2010) and by the H3K27 repressive chromatin modification. Based on these results, the population of Ncam1^+^ cells likely was heterogeneous, comprising a mix of stem and more committed progenitor cells.

We next determined whether hFK cultures would retain the NCAM1^+^ cells. For clinical applications in cell therapy, it is essential that cells can be maintained and expanded under defined serum-free conditions. We therefore cultured hFK cells in serum-free medium (SFM; Kreso & O'Brien, 2008) at low-density. Culturing in SFM generated compact colonies of small round/cuboidal cells after 14 days in culture, whereas culturing in serum-containing medium (SCM) yielded dispersed fibroblastic/spindle-shaped cells (Fig 1B). At the early stage of the isolation procedure serum may promote the selection and expansion of stromal lineages and also dedifferentiation via EMT (Ber et al, 2012; Xie et al, 2012), consistent with the observed morphology we observed. This was tested via gene and protein expression after one or two passages in SFM and compared to hFK cells cultured in SCM. We analysed a set of genes specifying undifferentiated mesenchymal renal progenitors (*OSR1*, *SIX2*, *PAX2*, *SALL1*, *WT1*), MET genes (*VIMENTIN*, *VIM*; *CDH1/E-CADHERIN*, *E-CAD*) and *FOXD1* which marks the stromal/interstitial lineage during renal development (Hatini et al, 1996; Humphreys & Bonventre, 2008). qRT-PCR analysis indicated that SFM cultures excluded stroma and deviated towards induced but immature epithelia and early post-MET state (Fig 1C). This was confirmed using antibodies to epithelial (EpCAM, CD24, CD133), mesenchymal (CD105, CD90) and haematopoietic/endothelial (CD34) cell surface markers (Supporting Information Fig S3A–C).

**Figure 2:**
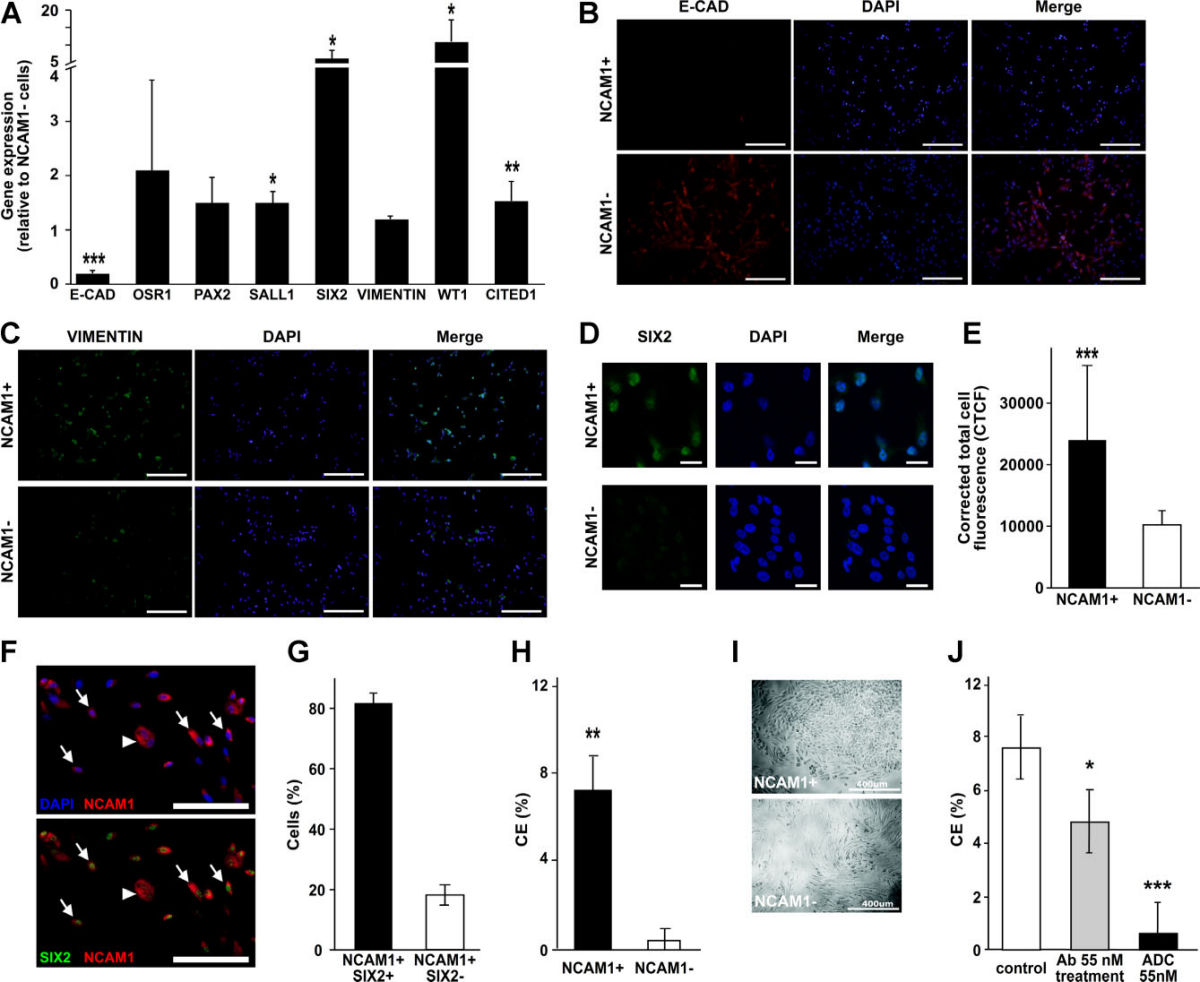
Characterization of immunosorted NCAM1^+^ subpopulation **A.** RT-PCR analysis of gene expression in NCAM1 cell fractions. *GAPDH* was used as endogenous control and NCAM1^−^ cell were used as the calibrator sample for RQ calculation (therefore = 1). Data were analysed using SDS 3.2 software and presented as average RQ ± SDEV of three replicates. ***p < 0.001, *p < 0.05 *versus* NCAM1^−^.**B–D.** Immunofluorescence staining of NCAM1^+^ and NCAM1^−^ subpopulations for E-cadherin (E-cad) (B, red) vimentin (C, green) and SIX2 (D, green). Nuclei stained with Dapi (blue). (B–C) Images were obtained using Olympus DP72 camera attached to Olympus BX51 fluorescence microscope and processed via cellSens standard software, bar represents 200 μm. (D) Images were obtained using Zeiss LSM 510 confocal microscope, bar represents 50 μm.**E.** Fluorescent quantification of SIX2 immunostaining as represented in (D).**F.** Double labelling of sorted NCAM1^+^ cells for NCAM1 and SIX2: NCAM1 with DAPI (upper panel; red and blue channels), NCAM1 with SIX2 (lower panel; red and green channels), indicating both NCAM1^+^ SIX2^+^ (arrows) and NCAM1^+^ SIX2^−^ (arrowheads) cells.**G.** Graph represents percentage of NCAM1^+^ SIX2^+^ cells and NCAM1^+^ SIX2^−^ cells.**H.** Clonogenic efficiency of NCAM1^+^ cells sorted from hFK and cultured in SFM. Data are presented as average CE(%) ± SDEV. **p < 0.01 *versus* NCAM1^−^.**I.** Representative morphology of NCAM1^+^ and NCAM1^−^ clones. Cells were observed using a Nikon Digital Sight camera attached to a Nikon Eclipse TS100 microscope.**J.** Clonogenic capacity of hFK cells treated with IMGN901(ADC 55 nM), huN901 (Ab55 nM) or not treated (control). Data are presented as average CE(%) ± SDEV. ***p < 0.001, *p < 0.05 *versus* control group.

Having established that hFK cells cultured in serum-free defined conditions skewed towards epithelial commitment but not uninduced mesenchyme or stromal lineage, we reasoned that derivation of NCAM1^+^ cells from hFK stroma where expression is abundant (Metsuyanim et al, 2009) would be eliminated while NCAM1 expressing cells originating from the nephrogenic pathway retained. Accordingly, culture and growth of hFK cells in SFM yielded a smaller NCAM1 expressing population (range 5–20% in SFM; Fig 2D and E) compared to SCM (>30% in SCM as described previously; Metsuyanim et al, 2009).

### Cultured NCAM1^+^ cells display essential stem/progenitor properties

We next determined whether the cultured NCAM1^+^ cells harbour stem cell characteristics. hFK cells were therefore expanded in SFM and NCAM1-positive cells were collected via immunosorting (Supporting Information Fig S3D and E) and analysed for the expression of nephron stem/progenitor cell markers. We utilized at least three human foetal samples that carry inherent variance and may hamper statistical significance. In the NCAM1^+^ cell population, we found significant elevation of the nephron progenitor markers compared to NCAM1^−^ cells (Fig 2A). Since NCAM1 is not detected in the UB epithelial lineage, markers such as PAX2 which strongly express in UB and derivatives were less enriched. Importantly, analysis of E-CAD levels showed significant reduction in NCAM1^+^ cells, indicating that under SFM conditions these cells resemble earlier stages of induction towards mature nephrons than the NCAM1^−^ sub-population. NCAM1^+^ immunosorted cells expanded in SCM showed less enrichment in nephron progenitor gene expression (Supporting Information Fig S3F). Expression analysis of NCAM1^+^ cells with oligonucleotide microarrays that allow a broader view of transcriptional changes (Supporting Information Table S1) showed up-regulation of multiple genes that positively regulate cell cycle/cell division/cell growth, concomitantly, with a reduction in genes associated with terminal epithelial differentiation (Supporting Information Table S2). Analysis at the protein level supported gene expression; immunofluorescent labelling of VIM and E-CAD revealed lack of E-CAD and positive VIM staining in NCAM1^+^ cells, whereas NCAM^−^ cells showed wide spread E-CAD expression (Fig 2B and C). In addition, SIX2 immunostaining demonstrated enhanced expression in NCAM1^+^ cells (Fig 2D–G, Supporting Information Fig S4) revealing the presence of earlier NCAM1^+^ SIX2^+^ characteristically displaying bright nuclei and small cytoplasm and more differentiated NCAM1^+^ SIX2^−^ cell populations (Fig 2F). Thus, we confirmed the existence of SIX2^+^ and SIX2^−^ sub populations in NCAM1^+^ cells from hFK samples observed in the mouse embryonic kidney cultures (Fig 1A).

Clonogenic capacity of NCAM1-positive and -negative cells was analysed by limiting dilution. When cells were plated under defined SFM conditions, clonogenic activity of hFK cells was found almost exclusively within the NCAM1^+^ fraction (Fig 2H and I). Self-renewal capacity of NCAM1^+^ cells was determined; cells were able to expand from one cell with no supporting cells to a clone that continued to expand up to three to four passages post-sorting maintaining a stable phenotype (Supporting Information Fig S5A). Hence, nephron precursors, although harbouring propensity for self-renewal, do not undergo endless self-renewing divisions under these culture conditions. To further improve expansion of NCAM1^+^ cells in SFM, we examined the addition of niche factors that have been shown to support mice nephron progenitors *in vitro* (Dudley et al, 1999). Only BMP7 in the absence of fibronectin increased cell numbers/survival by 50% on average, but activating WNT signalling failed to show a beneficial effect (Supporting Information Fig S5B and C).

Having established that NCAM1 enriches for clonogenic and progenitor properties of hFK cells, we determined whether specific targeting of the NCAM1^+^ fraction would deplete these properties. We therefore studied the effects of an antibody drug conjugate (ADC), which targets and depletes NCAM1 expressing cells (IMGN901; humanized NCAM1 monoclonal antibody covalently linked to the cytotoxic agent DM1; Lutz & Whiteman, 2009; Pode-Shakked et al, 2013) on clonogenicity/self-renewal capacity of hFK cells. Calibration assays were performed to determine optimal ADC dosing, thus enabling the elimination of the largest possible NCAM1^+^ cell fraction from hFK cells without harming other cells (Supporting Information Fig S5D and E). hFK primary cultures expanded in SFM were treated with 55 nM IMGN901, while control samples were given huN901 (NCAM1 antibody without the DM1 toxin) or left untreated. After 7 days of treatment, IMGN901-treated cultures were nearly abolished of clonogenic capacity and clone failed to propagate (Fig 2J), confirming that the stem/progenitor activity is contained in the NCAM1^+^ sub-population.

### *In vitro* cultured NCAM1^+^ cells retain *in vivo* nephrogenic potential

**Figure 3:**
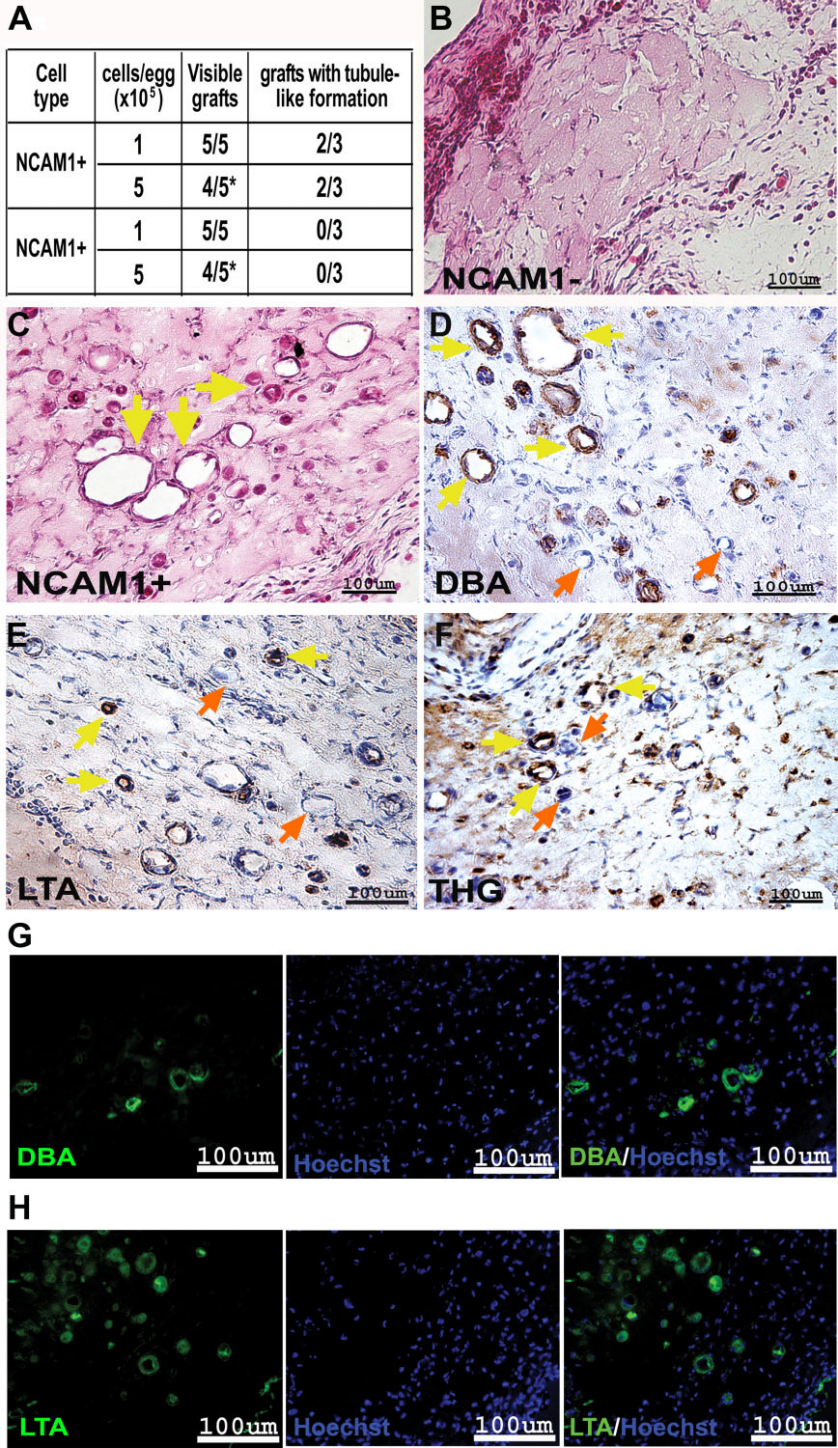
Segment-specific differentiation of NCAM1^+^ cells on chick CAM **A.** Summarizing table of chick CAM results (cell type, cells/egg, visible grafts and grafts with tubule-like formations).**B,C.** H&E staining of paraffin-embedded sections of 5 × 10^5^ NCAM1^−^ (B) and 5 × 10^5^ NCAM1^+^ (C) cells. Yellow arrows mark the tubule-like formations. The images were obtained using Scion colour digital camera attached to an Olympus BX51TF microscope.**D–F.** Segment-specific IHC staining of DBA (D), LTA (E) and THG (F) in paraffin-embedded sections of 5 × 10^5^ NCAM1^+^ cell grafts. Yellow arrows mark the stained tubule-like structures while orange arrows mark the unstained structures. Images were obtained using a Scion Corporation grayscale digital camera attached to an Olympus BX51.**G,H.** Segment-specific IF staining of DBA (G) and LTA (H) in paraffin-embedded sections of 5 × 10^5^ NCAM1^+^ cell grafts. Hoechst 33342 (blue) was used for nuclear staining. Images were obtained using an Olympus AX70 motorized microscope and spectral unmixing which allows distinction of background from specific staining, using a multispectral imaging system (NuanceFX camera and software).

To test the *in vivo* differentiation/organogenic capacities of SFM cultured NCAM1^+^ cells we performed grafting experiments on the chorio-allantoic membrane (CAM) of a chick embryo, a highly vascularized site supplying developmental cues (Buzhor et al, 2011; Noiman et al, 2011). We have previously established this as a model to analyse the ability of human kidney cell types to generate renal structures; non-renal cell types such as bone marrow mesenchymal stem cells (MSCs) have consistently failed (Buzhor et al, 2011; Noiman et al, 2011). No glomerular structures are apparent in CAM grafts, as their complex assembly is not likely to be supported in the CAM assay. In this assay, testing with different numbers of grafted human foetal cells showed that only high cell numbers of unselected hFK cells (≥2.5 × 10^6^) could generate tubule-like structures (Supporting Information Fig 6A–E). When 1 × 10^5^–5 × 10^5^ NCAM1^+^ were grafted we found identifiable tubular structures in 4/6 NCAM1^+^ cell grafts (an order of magnitude compared to unselected cells), while same numbers of NCAM1^−^ cells completely failed to generate similar structures (0/6 grafts; Fisher exact test, *p* = 0.03; Fig 3A–C). The human origin of hFK tubular structures on the CAM was confirmed by non-cross reactive human-specific Ki-67 antibodies, also indicating proliferative capacity of formed tubules (Supporting Information Fig S6F–H). Additionally, the epithelial marker panCK intensely stained the tubular structures but not individual cells yet to differentiate and organize (Supporting Information Fig S6I–K). We further analysed tubular structures derived from NCAM1^+^ cells for the presence of mature nephron cell types using markers specific for proximal (LTA; lotus tetragonolobus lectin), loop of Henle (anti-THG; Tamm–Horsfall glycoprotein) and distal (DBA; dolichos biflorus agglutinin) tubules. Immunostaining with LTA, DBA and anti-THG revealed the presence of all three (Fig 3D–H), which were devoid of NCAM1 expression (data not shown). Altogether, CAM grafting which enables the NCAM1^+^ progenitors to undergo MET into mature epithelia demonstrates their enriched and broad intrinsic differentiation capacity that NCAM^−^ counterparts lack.

### *In vitro* cultured NCAM1^+^ hNPCs improve the outcome of chronic renal injury

**Figure 4:**
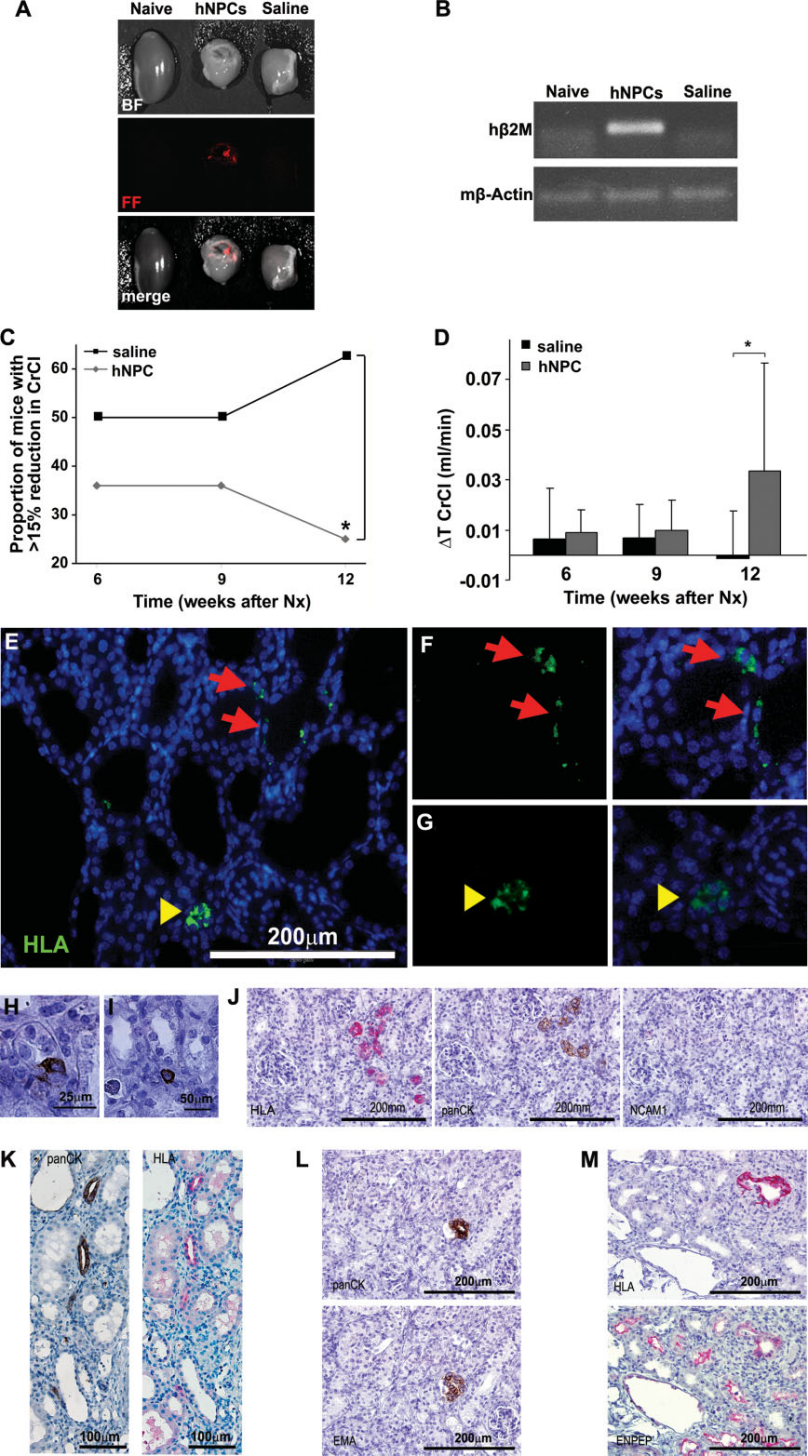
hNPC treatment in the 5/6 Nx chronic renal injury model **A,B.** hNPCs engraft into 5/6 Nx mouse kidneys: (A) hNPCs were labelled with CM-Dil Cell Tracker and injected directly into the renal parenchyma. Mice injected with saline served as controls. Two weeks after cell injection the kidneys were analysed by the maestroCRI *in vivo* imaging system. Shown are bright field (BF), fluorescence field in red (FF) and merged images of a healthy kidney (naïve, left), remnant kidney treated with hNPCs (middle) or with saline (right). (B) RT-PCR for human-specific β2-microglobulin (*hβ2m*) demonstrates a representative gel electrophoresis with amplification in the hNPC-injected kidneys, but not in untreated (naïve) or saline-injected kidneys. Mouse β-actin (*mβ-actin*) served as endogenous control.**C,D.** hNPCs halt disease progression and improve CrCl: (C) Injection of hNPCs into 5/6 Nx mice led to a significant decrease in the proportion of mice with >15% of renal function deterioration, compared to saline-treated mice. (D) hNPC-treated mice show a significant positive change in CrCl values at 12 weeks when compared to baseline levels (Δ*T* CrCl) in contrast to saline-treated mice. *p-Value <0.01.**E–M.** hNPCs integrate into existing tubules and regenerate new tubules: Injected remnant kidneys were stained for the human HLA marker (E–G, J, K—upper panel and M) and a human-specific epithelial marker, panCK/MNF116 (H–L). hNPCs integrated into tubules of host mouse (arrows) and regenerated new tubule-like structures (arrowheads), as demonstrated by IF HLA staining (E). Higher magnification of e is shown in (F and G) HLA staining (green, left) merged with DAPI (overlay, right). (H and I) Various modes of engraftment are highlighted: integration within tubules (H; panCK), integration between tubules (I; panCK) and tubular regeneration (J–M representing consecutive sections). (J) Small early tubules stained for HLA and panCK but not for NCAM1. (K) Mature tubules stained for HLA and panCK. (L) A human panCK expressing tubule (upper image) is further delineated to express the human-specific distal marker, EMA (lower image) (M) Staining for HLA (upper image) and cross-reactive proximal marker ENPEP (lower image) shows human origin of a regenerating proximal tubule type.

The data presented so far convinced us that the SFM cultured NCAM1^+^ cell population represents committed human nephron progenitors (hNPCs). To demonstrate the clinical importance of hNPCs, we carried out proof-of-principle experiments evaluating functional renal parameters in a mouse model of renal injury. *De novo* generation of mature renal cell types is likely to be relevant in chronic progressive kidney disease (*e.g*. chronic proteinuric states), in which nephron cells are progressively depleted. We therefore tested the therapeutic effects of hNPCs in the 5/6 nephrectomy (Nx) chronic progressive kidney injury model. We generated 5/6 Nx in 44 NOD/SCID mice and implemented a protocol of three consecutive injections of 5 × 10^5^ hNPCs directly into the renal parenchyma (every 3 weeks) and followed treated mice (*n* = 20) for a 14-week period in comparison to untreated mice (*n* = 24, saline injected; Supporting Information Fig S7A). For confirmation of cell engraftment hNPCs were labelled with CellTracker and analysed 2 weeks after cell injection using an *in vivo* imaging system (Fig 4A) and laser scanning confocal microscopy (Supporting Information Fig S7B). These data, along with the gene expression of human-specific β2-microglobulin (hβ2M) in the diseased murine kidney (Fig 4B, Supporting Information Fig S7C), indicated robust engraftment of hNPCs. The prolonged 5/6 Nx experiment enabled 24 h urinary collection and repeated measurement of creatinine clearance (CrCl) as a measure of renal function. For each time point we analysed the change from baseline CrCl (1 week post 5/6 Nx) among individual mice. At 12 weeks, the proportion of mice with a reduction of more than 15% in CrCl including mortality events was significantly lower in the hNPC treated group (5/20 mice) compared to the untreated saline group (15/24 mice; Fisher exact test, *p* = 0.01, Fig 4C). In addition, at 12 weeks but not in earlier time points, there was significant improvement in change from baseline in the absolute CrCl values with mean differences per minute of 0.034 ± 0.04 ml in the hNPC group *versus* −0.0137 ± 0.019 ml/min in the untreated group (*t*-test, *p* = 0.01; Fig 4D). Thus, treatment with hNPCs halted disease progression and increased CrCl throughout the 12-week study period, a finding that is consistent with an improvement in kidney function.

To further provide possible mechanistic insight as to the effects of hNPCs via production of renal epithelium and cell replacement we immunostained consecutive sections injured kidneys with human-specific markers, HLA and panCK/MNF116 (Cohen et al, 2010; Eventov-Friedman et al, 2005 and Supporting Information Fig S7D showing non-cross reactivity and specificity for human kidney epithelia). Three patterns of engraftment were observed, including integration into an existing mouse tubule (Fig 4E, F and H), between tubules (Fig 4I) and tubular regeneration (Fig 4E and G) consisting of small early tubules staining for HLA, panCK and lacking NCAM1 that appeared within 2 weeks (Fig 4J) and larger mature-appearing epithelial tubules with a patent lumen after 3 months (Fig 4K). Furthermore, immunostaining with distal (EMA) and proximal (ENPEP) markers disclosed renal identity (Fig 4L and Supporting Information Fig S7E; Fig 4M and Supporting Information Fig S7F, respectively). Altogether, repeated injection demonstrated that hNPCs have integrated to a certain extent into the endogenous mouse tissue, mixed with pre-existing host cells, and regenerating injured kidney tissue.

## DISCUSSION

Using defined culture conditions and NCAM1 immunosorting, we succeeded for the first time in prospectively isolating committed nephron progenitors from hFK. We showed that hNPCs have primitive characteristics that express distinct nephron progenitor markers. hNPCs also expressed relatively high levels of VIM and low levels of CDH1/E-CAD, corresponding to early stages of epithelial induction and confirming their primitive phenotype. Using clonal stem/progenitor cell assays, we found that NCAM1-expressing cells contain highly clonogenic and self-renewing cell populations (the latter to a limited extent in our culture conditions). Clonal hNPC were highly enriched for NCAM1 in SFM and NCAM1 targeting abrogated hFK cell clonogenicity. When grafted on the chick CAM these cells proved robustly tubulogenic *in situ* without the requirement of extra-stromal compartment and were more efficient at renal differentiation than were unsorted cells (whole hFK) and differentiated NCAM1^−^ counterparts. Importantly, foetal NCAM1^+^ cells not only exhibited rapid initiation of tubulogenesis but also broad nephron epithelial potential.

Previously, because of its mesodermal derivation, the hFK and undifferentiated MM were used as a valuable source of CD90^+^ plastic mesenchymal cells and MSC-like cells that gave rise to multiple lineages but not to kidney epithelial lineage (Almeida-Porada et al, 2002). Similarly, clonal progenitors isolated from the mouse embryonic kidney by means of selective growth of nephrospheres displayed broad mesodermal multi-potentiality (MSC-like) but not epithelial capacity and are thought to be derived from undifferentiated MM (Lusis et al, 2010). We obtained similar results when single cells derived from hFK were seeded at low density in SCM and spindle MSC-like cells potentially originating from undifferentiated MM or renal stroma emerged and became dominant in low passage. In fact, long-term self-renewing cell clones derived from our SCM cultures phenotypically lacked epithelial markers and resembled propagatable MSC populations commonly isolated by adhesion culture (Bruno et al, 2009; Dekel et al, 2006c). Importantly, in the chick CAM graft assay MSCs used as controls do not epithelialize to become nephron cell types (Buzhor et al, 2011). In contrast, when stimulated *in vitro* with serum-free defined conditions unsorted single hFK cells proliferated and readily acquired distinct morphology, inherent epithelial markers such as EpCAM and epithelial lineage commitment. NCAM1-expressing hNPCs, isolated from hFK cells expanded in SFM, are therefore clearly different both phenotypically and functionally from MSCs or MSC-like cells.

At present it is not possible to precisely identify the developmental stage the hNPCs originate from. Although the SFM culture conditions enrich the cultures as a whole for epithelial markers, the NCAM1^+^ sub-population appears to be less differentiated, as shown by their loss of CDH1/E-CAD and increase in SIX2 expression. During mouse kidney development expression of Cdh1/E-cad and Six2 was found to be almost mutually exclusive, with the exception of the cells that are going through the MET. One possibility is therefore that the hNPCs are this minor subset of E-CAD/SIX2^+^/NCAM1^+^ cells. It is however not clear if these cells are truly expressing both SIX2 and E-CAD, or the observed co-expression is the result of SIX2 protein stability after its expression has been stopped. Alternatively, the hNPCs could be the complete SIX2^+^ sub-population of the NCAM1^+^ cells and therefore be derived from the CM. Further fractionation of the NCAM1^+^ population in mouse and human may separate early (NCAM1^+^SIX2^+^) and more committed nephron progenitors (NCAM1^+^SIX2^−^) and indicate functional potency. This was recently evident in Wilms' tumour for which an additional marker (ALDH) within the NCAM1^+^ fraction was used to more precisely pinpoint cancer stem/initiating cells in the tumour (Pode-Shakked et al, 2013). We therefore, at this stage, collectively consider foetal human progenitors expressing NCAM1 as a pool of mitotically competent yet strongly nephron-generating-biased cells.

Neither haematopoietic stem cells (HSCs) nor MSCs, the most accessible human stem cells, can be used to derive stem/progenitor cells capable of generation of renal components (Almeida-Porada et al, 2002; Harari-Steinberg et al, 2011; Krause & Cantley, 2005; Pleniceanu et al, 2010). The ability of hNPC within their capacity to carry out developmental functions and unequivocally generating human renal structures encouraged us to investigate the possibility of injecting hNPCs to restore function in models of kidney failure (Harari-Steinberg et al, 2011; Pleniceanu et al, 2010). Importantly, we could show beneficial effects of hNPC on improved renal function in a mouse model of chronic progressive renal injury that mimics more closely CKD in humans. Since hNPC long-term engrafted in the 5/6 Nx chronic injury model to produce kidney epithelium (even at 3 months post-hNPC injection) demonstrating regenerative potential in the remnant kidney, we hypothesize that such increases from baseline CrCl adhere at least in part to epithelial lineage differentiation of hNPCs and functional restoration. Nevertheless, tissue stem/progenitors have potential to decrease inflammation and oxidative stress and to improve angiogenesis processes altered in CKD to create a proper renal microenvironment (Yasuda et al, 2010). With the level of integration observed delineation of the molecular mediators and functional effectors of hNPC is required to assess the relative contribution of differentiation-dependent and -independent mechanisms in CKD. Altogether, while our data suggest a reparative potency for hNPC and provides cautions optimism for proceeding with allogeneic renal stem/progenitor cell therapy (ideal for clinical scenario in which CKD patients are already subscribed with an immunosuppressive drug), the absence of a significant improvement in changed CrCl following the first and second injections of hNPC and the improvement observed at 10 weeks (after three injections) suggest that in the chronic scenario sustained improvement in CrCl may require repeated injections leading to cumulative effects of hNPCs rather than single administration. These results imply the anticipated use of large cell numbers and highlight the importance in developing tools for more robust clonogenic self-renewal of hNPC and *ex vivo* expansion. In this regard, BMP7 showed a positive effect on hNPC growth in accordance with data in mice (Blank et al, 2009; Dudley et al, 1999). Nevertheless, deciphering additional factors to further maintain committed nephron stem/progenitors (within the NCAM1^+^ fraction) is warranted. Finally, as congenital human kidney malformations are fundamentally disorders of the renal progenitor pool, cultured hNPCs with their ability to engraft and regenerate in hosts may provide new opportunities for functional analysis and disease modelling of novel inherited mutations leading to abnormal nephrogenesis and human renal developmental malformations (Vivante et al, 2013).

## MATERIALS AND METHODS

### Ethics statement

This study was conducted according to the principles expressed in the Declaration of Helsinki and was approved by the Institutional Review Boards of Sheba Medical Center, Wolfson Hospital and Asaf Harofeh Medical Center. All pregnant women involved in the study provided written informed consent for the collection of samples from their aborted foetuses and subsequent analysis.

### Establishment of a primary culture from human kidney

hFK were collected from foetuses that were aborted electively. Foetal gestational age ranged from 15 to 23 weeks. Collected tissues were washed with cold HBSS (Invitrogen) and minced into ∼1 mm cubes using sterile surgical scalpels. The dissected tissue was then incubated for 2 h in 37°C with Iscoves' Mod Dulbecco's Medium (IMDM; Invitrogen) supplemented with 0.1% collagenase IV (Invitrogen). The digested tissue was then gradually forced through 100, 70 and 50 µm cell strainers to achieve a single cell suspension. After the digesting medium was removed, the cells were resuspended in a growth medium (*either serum-containing or serum-free medium*) and plated in flasks. Serum containing medium (SCM) was comprised of IMDM (Biological Industries) supplemented with 10% foetal bovine serum (Invitrogen), 1% Pen–strep 100 M, 1% l-glutamine (both from Biological industries), 100 ng/ml EGF, 100 ng/ml bFGF and 10 ng/ml SCF (all growth factors purchased from Peprotech Asia). For passaging, cells were detached using 0.05% trypsin/EDTA (Invitrogen). Serum-free medium (SFM) was comprised of N2 medium (Biological Industries) supplemented with 1% Pen–strep 100 M, 1% l-glutamine, 0.4% B27 supplement (Gibco), 4 µg/ml heparin sodium (Intramed), 1% non-essential amino acids, 1% sodium pyruvate, 0.2% CD lipid concentrate (all from Invitrogen), 2.4 mg/ml glucose, 0.4 mg/ml transferrin, 10 mg/ml insulin, 38.66 µg/ml putrescine, 0.04% sodium selentine, 12.6 µg/ml progesterone (all from Sigma–Aldrich), 10 ng/ml FGF and 20 ng/ml EGF. For passaging, cells were detached using non-enzymatic cell dissociation solution (Sigma–Aldrich). The cells were observed using Nikon Eclipse TS100 and Nikon Digital Sight cameras.

### Immunoflorescence (IF) staining of mouse embryonic kidney organ cultures

Kidneys from E11.5 CD1 embryos were dissected and cultured on transwell membrane plates (Corning) for 4 days in Eagle's MEM (Sigma) with 10% FCS and pen/strep. Cultured kidneys were fixed in 4% PFA and antibody stained. Primary antibodies for Six2 (LifeSpan Biosciences), Cdh1 (BD transduction Laboratories) and Ncam1 (Sigma) and secondary antibodies used are summarized in Supporting Information Table S5. Images were obtained using Nikon A1R confocal microscope with and processed in ImageJ/Fiji software.

### IF staining of cells

Cells were fixed with 2% PFA with 3% sucrose in PBS (for SIX2 primary antibody) or with 4% PFA (for vimentin and E-cadherin) for 10 min, and washed with PBS. Cells were blocked with 5% human serum, 5% donkey serum and 1% BSA in PBS–Tween (0.05%) followed by incubation a primary antibodies for SIX2 (Novus), vimentin (Abcam) or E-cadherin (Cell Signaling) (see Supporting Information Table S5) for 1 h in room temperature. Cells were washed and then incubated with a secondary antibody for 1 h in room temperature. Mounting containing DAPI (Dapi Fluoromount-G; SouthernBiotech, 0100-20) was applied. Images were obtained by Olympus BX51 fluorescence microscope using Olympus DP72 camera and cellSens standard software.

For comparative analysis between the fluorescence signal of NCAM1 sorted cells, images were acquired using identical settings (exposure and gain setting) using Zeiss AxioVision Software Package and analysed using ImageJ software. Signal quantification was calculated for each nucleus by determining the nuclei area and quantization of the signal in that area. The corrected total cell fluorescence (CTCF) was calculated as: integrated density − (area of selected cell × mean fluorescence of background reading).

### Flow cytometry (FACS)

1 × 10^5^ Cells were suspended in FACS buffer (0.5% BSA, 2 mM EDTA in 1× PBS) and blocked with FcR blocking reagent (MiltenyiBiotec) and human serum (1:1). The cells were then incubated with a primary antibody or an isotype control (summarized in Supporting Information Table S3). Cells were incubated with a secondary antibody if needed (summarized in Supporting Information Table S4). Cell viability was tested using 7AAD viability staining solution (eBioscience). Cell labelling was detected using FACSCalibur (BD Pharmingen). FACS results were analysed using FlowJo analysis software.

### Magnetic cell sorting

Cultured hFK cells were collected, transferred through a 30 µm pre-separation filter (Miltenyi Biotec GmbH) and then washed and resuspended in pH 7.2 FACS buffer. Cells were magnetically labelled with CD56 (NCAM1) magneticbeads (Miltenyi Biotec GmbH) according to the manufacturer's instructions. Positive labelled cells were enriched on LS columns (Miltenyi Biotec GmbH) according to the manufacturer's instructions. Cell enrichment was validated using FACS.

### Comparative real-time RT-PCR analysis of gene expression

Total RNA was isolated using RNeasy micro or mini kits (Qiagen GmbH) according to the manufacturer's instructions. cDNA was synthesized using the high capacity cDNA reverse transcription kit (ABI). Real-time RT-PCR (qRT-PCR) was performed using the ABI7900HT sequence detection system (Perkin-Elmer/Applied Biosystems) in the presence of TaqMan Gene Expression Master Mix or Universal PCR Master Mix (both from ABI). qRT-PCR amplification was performed using TaqMan Gene Expression Assay using StepOne plus real time RT-PCR system (ABI). h*HPRT1* was used as endogenous control. The results were analysed using StepOne Software v2.1 in the ΔΔ*C*_T_ (such that the expression levels of the calibrator samples = 1). Statistical analysis was performed using a non-paired 2-tailed *t*-test. Statistical significance was considered to be *p*-value <0.05.

### Renal differentiation of cells on chick chorio allantoic membrane (CAM)

Fertile chicken eggs were obtained from a commercial supplier and incubated at 37°C at 60–70% humidity in a forced-draft incubator. hFK cells cultured in SFM (P2) were sorted to NCAM1^+^ and NCAM1^−^ subpopulations using magnetic beads, as described above. Cells were grafted on the CAM, as described previously (Buzhor et al, 2011; Noiman et al, 2011). On Day 9 or 10 of incubation, 1 × 10^5^ or 5 × 10^5^ cells/egg suspended in 50 µl Matrigel (BD Biosciences) were pipetted into an approximately 0.8 cm plastic ring placed on the CAM (five replicates). The eggs were then incubated for an additional 5–7 days. Cell grafts were photographed after removal using a Scion Corporation colour digital camera (model CFW-1612C) attached to an Olympus SZX12 (model SZX-ILLB200). Grafts were fixed overnight in 4% buffered paraformaldehyde and embedded in paraffin. Three grafts were sampled from each treatment group for haematoxylin and eosin (H&E) staining, and one was also used for immunohistochemical (IHC) and immuofluorescence (IF) staining.

**The paper explained**

PROBLEM:

The kidney is an essential organ in mammals, which removes waste products and blood and regulates intravascular volume, electrolyte and acid/base homeostasis. Kidney disease is increasing at high rates. At present, dialysis and transplantation remain the only treatment options. Nevertheless, the shortage of available organs for transplantation continues to severely limit the latter option. Thus, there exists a tremendous clinical need to generate/construct/repair nephrons, the functional filtration unit of the kidney, and improve renal function. Regenerative medicine relies on cell transplantation, materials science, and biomedical engineering to develop biologic substitutes that can restore and maintain normal function of damaged or lost tissues and organs. We and others have been investigating the possibility of injecting functional cells to induce renal regeneration in models of kidney failure. The first challenge is to identify a stem cell capable of generation of renal components. Neither haematopoietic stem cells (HSCs) nor mesenchymal stem cells (MSCs), the most accessible human stem cells, can be used to derive true renal progenitors. Given this lack of an easy source for renal stem cells, we looked for an alternative source.

RESULTS:

Formation of kidney tissue during embryonic development requires the generation of kidney precursor cells and their subsequent differentiation into functional nephron units. Therefore, a source for renal progenitor cells is the developing kidney. We therefore went on to identify stem/progenitor cells from within this tissue. Using defined serum free culture conditions that were adopted from studies on epithelial stem cells and specifically stimulate growth of the renal epithelial lineage and immunosorting according to NCAM1, we succeeded for the first time in prospectively isolating mitotically active human epithelial nephron progenitors (hNPCs). We show that hNPCs have primitive characteristics expressing distinct nephron progenitor markers and are highly clonogenic. Moreover, when grafted in aggregates onto the chorioallantoic membrane (CAM) of the chick embryo these cells proved robustly to generate renal structures. Collectively, foetal NCAM^+^ cells exhibited broad nephron epithelial differentiation (proximal, loop of Henle and distal tubules (data not shown), while in contrast grafting of control MSCs leads to generation of a disorganized cell mass. Finally we show the beneficial effects of hNPCs in a mouse model of chronic renal injury that mimics a common clinical condition in humans harboring chronic kidney disease (CKD).

IMPACT:

The identification of hNPCs represents advancement in stem cell science and renal medicine and paves the way to developing novel renal regenerative therapies in humans.

### H&E staining of paraffin-embedded CAM grafts

Six micrometre sections of paraffin-embedded CAM grafts were mounted on super frost/plus glass and incubated at 60°C for 40 min. After deparaffinization, slides were incubated in Mayer's haematoxylin solution (Sigma–Aldrich) and incubated with 1% HCl in 70% ethanol for 1 min. Slides were then incubated for 10 s in eosin (Sigma–Aldrich). Images were produced using Olympus BX51TF and Scion colour and monochrome digital cameras.

### IHC and IF staining of paraffin-embedded CAM grafts

Graft sections were mounted on super frost/plus glass, as described above, and deparaffinized. After 15 min of incubation in 3% H_2_O_2_ in methanol, antigen retrieval (only in THG staining) was performed by boiling the slides for 15 min in a 0.01 M sodium citrate buffer (using a microwave oven). Slides were then blocked with 1% BSA in PBS for 15 min and incubated overnight at 4°C with a primary antibody (see Supporting Information Table S5). In the negative control samples, the primary antibody was substitute with non-immune serum. When IHC staining was carried out, a biotinylated second antibody was applied for 1 h, followed by incubation with horseradish peroxidase-conjugated streptavidin (HRP-SA) for 10 min. The immunoreaction was visualized by an HRP-based chromogen/substrate system, including DAB (brown) chromogen (liquid DAB substrate kit—Zymed) according to the manufacturer's instructions. The sections were then counterstained with Mayer's haematoxylin solution, dehydrated and mounted for microscopic examination. Images were obtained using a Scion Corporation grayscale digital camera (model CFW-1312M) attached to an Olympus BX51 (model BX51TF). When IF staining was performed slides were incubated for 1 h with a secondary antibody (see Supporting Information Table S5) at room temperature and counterstained with 0.1 µg/ml Hoechst in PBS for 5 min. Slides were then coverslipped in an anti-fade medium [90% glycerol (Sigma–Aldrich) with 10% PBS and 1% *n*Propyl gallate (Sigma–Aldrich)]. Images were obtained using an Olympus AX70 motorized microscope, and spectral unmixing using a multispectral imaging system (NuanceFX camera and software) was applied, as described previously (Noiman et al, 2011) due to the strong autofluorescence present in both the grafted tissue and the hFK sections.

### 5/6-Nephrectomy model of chronic renal disease in NOD-SCID mice

Mice at 9–12 weeks of age were subjected to two-step 5/6 nephrectomy under halothane anaesthesia (Kasiske et al, 1988; Kennedy et al, 2003). This model was used to evaluate the therapeutic properties of hNPCs. One week after the second session, 5 × 10^5^ cells were injected directly into the parenchyma of the remnant kidney in three separate injections at intervals of 3 weeks between injections. Two weeks after each cell injection, 24 h urine samples were collected using metabolic cages and blood samples were collected from the retro-orbital sinus. On the 10th week mice were fed by high protein food followed by a fourth blood and urine sample collection. Fourteen weeks after the second Nx session, the mice were sacrificed and the kidneys were removed for IHC analysis. CrCl in ml/min was calculated using the following formula:


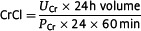


### IHC and IF staining of paraffin-embedded human and mouse kidney tissues

Sections were pre-treated using OmniPrep solution (Zytomed Systems) in 95°C for 1 h according to manufacturer's protocol. Blocking was performed using Cas-Block solution (Invitrogen) for 20 min followed by 1 h of incubation with a primary antibodies (Cytokeratin, HLA, EMA, EnPEP or NCAM1; see Supporting Information Table S5). Samples were incubated in secondary antibody (see Supporting Information Table S5) for 30 min in room temperature and detected using DAB kit (Cell Marque) or Red Substrate Kit (Vector), both according to manufacturers' protocols. Haematoxylin was used for counterstaining. For IF staining, sections were blocked with 5% human serum, 5% donkey serum and 1% BSA in PBS-Tween (0.05%) for 1 h followed by incubation with the primary antibody for HLA (Supporting Information Table S5) and then washed and incubated with secondary antibody (Supporting Information Table S5) for 1 h. Mounting containing DAPI was applied.

For more detailed Materials and Methods see the Supporting Information.

## Supplementary material

As a service to our authors and readers, this journal provides supporting information supplied by the authors. Such materials are peer reviewed and may be re-organized for online delivery, but are not copy-edited or typeset. Technical support issues arising from supporting information (other than missing files) should be addressed to the authors.

**Figure S1. Gene expression coordinately regulated with NCAM1 during mouse kidney development.** HeatMap of gene expression in different renal compartments and time points during mouse kidney development and adulthood, that their expression was coordinately regulated with NCAM1 expression. Genomic data was retrieved from the GUDMAP database (Harding et al, 2011) and analysed using Pearson correlation assay across the normalized gene expression profiles.

**Figure S2. NCAM1 expression module.** A scheme of the NCAM1 module network. Genomic data was retrieved from GUDMAP database (Harding et al, 2011) and analysed using Toppcluster (Kaimal et al, 2010). Genes in the NCAM1 expression module that are known to play a role in neurogenesis at some level are highlighted.

**Figure S3. Characterization of hFK cells (A) FACS analysis of surface marker expression in hFK cell cultures.** Each marker was tested on at least three independent samples. Data was calculated as the average percentage of expressing cells ± SDEV. (B and C) Immunofluorescence staining of hFK cells cultured with SFM for CD24 (B, red) and EpCAM (C, green). Nuclei were stained with Dapi (blue). Images were obtained using Olympus DP72 camera attached to Olympus BX51 fluorescence microscope and processed via cellSens standard software. (D and E) Isolation of NCAM1^+^ cells from hFK cells. Representative FACS sorting analysis of NCAM1 expression in hFK cells cultured in SFM at passage #1. Data are presented in histogram graphs, mean fluorescence intensities (MFI) and percentage of NCAM1 expressing cells. Shown are NCAM1 (right panel) and isotype control (left panel) staining (D). (E) Validation of sorting purity of NCAM1^−^ (left panel) and NCAM1^+^ (right panel) sub-populations. (F) Characterization of NCAM1 cells sorted from SCM cultures. qRT-PCR analysis of gene expression in NCAM1 cell fractions grown in SCM. Normalization was performed against control *GAPDH* expression and RQ calculated relative to NCAM1^−^ cell fraction. Data were analysed using SDS 3.2 software and presented as average RQ ± SDEV of three replicates. ****p* < 0.001, **p* < 0.05 *versus* NCAM1.

**Figure S4. Calibration of immunoflorescence staining of SIX2.** Double IF staining of SIX2 (green) and NCAM1 (red) in hFK NCAM1^+^ (B) and NCAM1^−^ (D) sorted cells. Cells from primary cultures of Wilms tumour (A) or adult kidney (D) were used as positive or negative controls (respectively) of the SIX2 staining. Nuclei were stained with Dapi (blue). Images were obtained using Zeiss SLM 510 microscope and processed in ImageJ/Fiji software. Scale: 50 µm.

**Figure S5. *In vitro* characterization of NCAM**^**+**^ **cells.** (A) Representative graph of self-renewal in NCAM1^+^ cells sorted from hFK that was cultured in SCM. Data are presented for each passage as relative number of clones developed from the total number of cells plated. (B) Percentage of surviving NCAM1^+^ cells after sorting and culturing with or without BMP7 for 7 days. Each treatment was tested on three independent samples. Number of cells was calculated as percentage of the control group and presented as average of three replicates ± SDEV. (C) Percentage of surviving NCAM1^+^ cells after sorting and culturing with or without BIO. Number of cells was calculated as percentage of the control group. (D) Proliferation analysis of hFK cells treated with different dosages of IMGN901 (0–1.675 μM) as demonstrated by MTS proliferation assay. OD levels are presented as percentage of the control sample (0 μM). (E) FACS analysis of the NCAM1 population in hFK treated with different dosages of IMGN901 (0–55 nM). Results are presented as percentage of the control sample (0 μM). FIB, fibronectin coating.

**Figure S6. Renal differentiation of hFK cells on chick CAM.** (A) hFK cells grafted on the chick CAM. Graft images were obtained using Scion Corporation colour digital camera attached to Olympus SZX12. (B) Summarizing table of the amount of hFK cells grafted on the CAM (grafted cells, replicates, visible grafts and grafts with tubule-like formations). (C–E) H&E staining of paraffin embedded sections of 0.35 × 10^6^ (C), 1.25 × 10^6^ (D) and 2.5 × 10^6^ (E) grafted cells. Images were obtained using Scion colour digital cameras attached to Olympus BX51TF microscope. (F–K) IF staining of Ki67 (F–H, red) and pan-CK (I–K, green) in paraffin embedded sections of 2.5 × 10^6^ cell grafts. Hoechst 33342 (Blue) was used for nuclear staining. Images were obtain using Olympus AX70 motorized microscope and spectral unmixing using multispectral imaging system (NuanceFX camera and software).

**Figure S7. Chronic renal disease model of 5/6-Nephrectomy (5/6 Nx) in NOD/SCID mice.** (A) Schema of *in vivo* experiments: 5/6 Nx in NOD/SCID mice was performed in two steps (Nx1-unilateral nephrectomy and Nx2-2/3 nephrectomy). One week after the second step, hNPCs were injected directly into the remnant kidney (transplantation #1–3) in three cycles, with 3 weeks intervals between each injection. Blood and urine were collected 2 weeks after each cell injection (sample collection #1–3). Starting on the 10th week mice were fed by high protein food followed by another blood and urine sample collection (#4). Fourteen-week after the second step of the Nx the mice were sacrificed and the kidneys were removed for IHC or gene expression analysis. (B) hNPCs were labelled with CM-Dil Cell Tracker Mice injected with saline served as controls. Sections of the injected kidneys were then analysed by confocal microscopy. While saline injected kidney show no detectable signal, hNPCs are seen in the injected kidneys as soon as 24 h after the injection and are still detectable at 2 weeks. (C) RT-PCR for human-specific β2-microglobulin (*hB2m*) was performed to validate the presence of human cells in the injected mouse kidney. Representative real-time PCR amplification plot demonstrates amplification in the hNPC-injected mouse kidneys, but not in untreated (naïve) or saline-injected kidneys. Mouse β-actin (*mβ-actin*) served as endogenous control. (D) IHC with human specific antibody for pan-cytokeratin/MNF116 is shown in positive control hFK tissue (left panel), and in two negative control NOD/SCID kidney tissues: healthy/naive mouse kidney (MK-naïve, middle panel) and in 5/6 Nx kidney injected with saline (MK-5/6 saline, right panel). (E) IHC with human specific antibody for EMA in MK-5/6 saline (right panel) and in positive control human adult kidney (AK) tissue (right panel). (F) IHC with human antibody for ENPEP is shown in both MK-5/6 saline (left panel) and AK (right panel) tissues. Bar represents 200 μm.

**Table S1.** Expression analysis of NCAM1^+^ cells with oligonucleotide microarrays.

**Table S2.** Differential expressed genes in NCAM1^+^ cells.

**Table S3.** Primary antibodies used in FACS assays.

**Table S4.** Secondary antibodies used in the FACS assays.

**Table S5.** Antibodies used in IHC or IF staining..
