# Supplementary material for: Identification of human nephron progenitors capable of generation of kidney structures and functional repair of chronic renal disease
Source: EMBO Mol Med. 2013 Sep 2;5(10):1556–68. doi: 10.1002/emmm.201201584 (PMC3799579; doi:10.1002/emmm.201201584)
Supplement: Supplementary file 1 [file emmm0005-1556-SD1.pdf]

EMBO Molecular Medicine

Identification of human nephron progenitors capable of generation of kidney structures and functional repair of chronic renal disease

Orit Harari-Steinberg, Sally Metsuyanim, Dorit Omer, Yehudit Gnatek, Rotem Gershon, Sara Pri-Chen, Derya Deniz Ozdemir, Yaniv Lerenthal, Tzahi Noiman, Herzel Ben-Hur, Zvi Vaknin, David F. Schneider, Bruce J. Aronow, Ronald S. Goldstein, Peter Hohenstein and Benjamin Dekel

Corresponding author: Benjamin Dekel, The Pediatric Stem Cell Research Institute, Edmond & Lily Safra Children's Hospital, Sheba Center for Regenerative Medicine, Sheba Medical Center, Israel.

Review timeline:

Submission date:	22 May 2012
Editorial Decision:	25 June 2012
Revision received:	17 January 2013
Editorial Decision:	05 March 2013
Revision received:	06 June 2013
Editorial Decision:	08 July 2013
Revision received:	17 July 2013
Editorial Decision:	19 July 2013
Revision received:	29 July 2013
Accepted:	31 July 2013

Transaction Report:

(Note: With the exception of the correction of typographical or spelling errors that could be a source of ambiguity, letters and reports are not edited. The original formatting of letters and referee reports may not be reflected in this compilation.)

Editor: Céline Carret

1st Editorial Decision 25 June 2012

Thank you for the submission of your manuscript to EMBO Molecular Medicine. We have now heard back from the three referees whom we asked to evaluate your manuscript. As you will see from the reports below, the referees find the topic of your study of potential importance. However, they raise substantial concerns on your work, which should be convincingly addressed in a major revision of the present manuscript.

Indeed, all three reports are very consistent and claim a lack of statistical significance (sample numbers, error bars, etc), lack of protein expressions to substantiate the findings, unsufficient experimentations to support firmly some of the conclusions made. The reports are quite explicit, and to address all issues satisfactorily, some extensive and lengthy revision might be required.

We would be willing to consider a revised manuscript with the understanding that all the referees' concerns must be fully addressed and that acceptance of the manuscript would entail a second round of review.

I should remind you that it is EMBO Molecular Medicine policy to allow a single round of revision only and that, therefore, acceptance or rejection of the manuscript will depend on the completeness

of your responses included in the next, final version of the manuscript. I realize that addressing the referees' comments in full would involve a lot of additional experimental work and I am uncertain whether you will be able (or willing) to return a revised manuscript within the 3 months deadline and I would also understand your decision if you choose to rather seek rapid publication elsewhere at this stage.

Revised manuscripts should be submitted within three months of a request for revision; they will otherwise be treated as new submissions, except under exceptional circumstances in which a short extension is obtained from the editor. Also, the length of the revised manuscript may not exceed 60,000 characters (including spaces) and, including figures, the paper must ultimately fit onto optimally ten pages of the journal. Should you find the length constraints to be a problem, you may consider including any peripheral data (but not methods in their entirety) in the form of Supplementary information.

I look forward to seeing a revised form of your manuscript as soon as possible.

Should you find that the requested revisions are not feasible within the constraints outlined here and choose, therefore, to submit your paper elsewhere, we would welcome a message to this effect.

***** Reviewer's comments *****

Referee #1 (Comments on Novelty/Model System):

The mouse is a tractable and affordable model in which to test therapeutic engraftment of human tissue. For translational purposes, testing in additional models will be required, but for the proof of principle experiment described in this paper a rodent model is adequate.

Referee #1 (Other Remarks):

The authors have generated an isolation and culture system for nephron progenitor cells from embryonic human kidney tissue. They have used this method to derive kidney cells displaying gene expression characteristics of nephron progenitors, and have subsequently engrafted these cells into two model systems: i) Chick chorioallantoic membrane, ii) Mice with glycerol induced acute kidney injury. They find that the progenitor cells form epithelial structures in the chick assay, and that there is some protective effect of engraftment in the mouse model of kidney injury. Alternative sources of kidney tissue is an active area of research because of the clinical need, and the work is potentially significant. Although the authors have conducted a thorough and convincing analysis of the purified cells, the novelty and impact of the paper rest on the finding that injected cells are renoprotective in a model of acute kidney injury, which is the weakest experiment in the manuscript. Several issues surrounding the interpretation of results and choice of model need to be clarified to substantiate the authors' conclusions:

1. There is no lineage analysis (human vs chick) in the chick experiment showing the origin of the epithelial structures that arise following engraftment. This is a standard proof for an engraftment experiment and it should be included.
2. The authors do not show engraftment of injected cells into the kidney, they simply show that Qdot labeled cells localize to the kidney. Trapping of cells and possibly free Qdots in the fine vasculature of the kidney is anticipated, so simply showing a low magnification shot of Qdot signal in the organ is not convincing - the authors need to evaluate histologically if Qdot labeled cells are incorporated into kidney structures to see if there is tissue engraftment, or if Qdots and cells are merely trapped. A control showing that Qdot-loaded particles the size of a cell, or unrelated cells loaded with Qdots, do not stick in the kidney would be a welcome addition to this dataset.
3. Some of the earliest experiments done to assess the renoprotective effects of stem cells were confounded by the fact that the cells produce renoprotective factors that are secreted into the culture medium. Thus, removing the cells from the engraftment mixture did not significantly reduce the renoprotective effect of the "engraftment". Lloyd Cantley's group has published several studies on this phenomenon, which may be very significant clinically. To support the last sentence of the

abstract, it would be important to know whether the NCAM+ cells are exerting their renoprotective effect through cellular engraftment or production of soluble renoprotective factors.
3. The choice of injury model is confusing because the ATN following glycerol injection is secondary to muscle injury. No clinical chemistry is provided to prove that the cell injection is not protecting the mice from rhabdomyolysis, with a secondary ameliorative effect on kidney injury.
4. The use of a single parameter to judge kidney injury is insubstantial. Histological evaluation should be included.

Additional points:
qPCR is not be the best way to tell if cells in the NCAM fraction are enriched for epithelial progenitors. Six2 is likely expressed by other cells at lower levels and maybe these are eliminated in the NCAM positive fraction. SIX2 co-stain on the NCAM fraction would be conclusive.

DBA lectin is an odd choice to mark distal tubule as it is generally used to mark collecting duct quite specifically in the mouse (Laitinen et al. 1987. Changes in the glycosylation pattern during embryonic development of mouse kidney as revealed with lectin conjugates. J Histochem Cytochem 35:55-65). If DBA marks distal tubule in the human kidney, the authors should provide a reference. If not, it would mean that structures of collecting duct identity develop from the NCAM+ fraction, which would either indicate contamination of the cell preparation used for engraftment, or that cells of the nephron lineage have transdifferentiated to the collecting duct lineage in this assay.

It seems bold to say in the abstract that human nephron progenitor cells prevent death and renal failure when all that was observed was a trend. If the authors want to make this claim they need to analyze an appropriate number of mice based on a power analysis of their current data.

Referee #2 (Other Remarks):

This manuscript describes the isolation of a population of cells from human fetal kidneys that can be expanded in vitro, have characteristics of nephron progenitor cells and when transplanted on chick chorio allantoic membrane can give rise to structures that express nephron specific markers. Moreover, the authors performed experiments in which transplantation of NCAM+ cells provide some protective effect from glycerol induced acute renal failure.

The isolation and identification of a human nephron progenitor cells is an important goal, as it may in the long run help treating renal diseases. The manuscript by Metsuyanim et al., provides some important steps towards achieving this goal. Unfortunately, several experiments are incomplete and a number of issues need to be resolved before I can support publication.

1.The first part of the paper describes the culture and characterization of minced human embryonic kidney in serum free and containing medium. Figure 1 shows quantitative analysis of several genes expressed within the developing kidney, but I am wondering how strong the expression is, when compared to wildtype developing kidneys/cap mesenchyme. Also, I don't understand why no error bars are shown for SCM samples. I realize that the value for the SCM cultures was set to one, but if 3 independent samples have been used (figure legends), I would expect to have some variability between the individual samples.

2.Limited diluted cells expanded in SCM form clones, but appear to undergo a crisis at about passage 5, a process that is often observed in primary cultures. In the following analysis only 2 independent clones appeared to be analysed. Since the variation of expression of these two clones is quite substantial, I am not sure one can draw clear-cut conclusions. This is even more worrying given substantial variability of gene expression in clone 1 and 2 for several of the genes analysed (CD90, CD105, Foxd1, Sall1, vimentin...). I realize that human material is limited, but given the low sample number, I am somewhat concerned regarding the reproducibility of the data?

3.Transplantation experiments into the chick chorio allantoic membrane suggest that NCAM+ cells can develop into epithelial structure that express markers of various nephron segments and the authors conclude that the transplanted cells have undergone differentiation into nephrons. However,

NCAM positive cells are not restricted to the cap mesenchyme (progenitor compartment), but can also be found within epithelial comma- and S-shaped bodies (Supp. Fig1). It is not clear to me whether the epithelial cells detected after transplantation are derived from mesenchymal cells (differentiation) or whether they represent epithelial cells already present in the original NCAM+ population (and maybe reassembled as tubules). Do the authors have a way to address this question?

4.My biggest problem with this paper relates to the in vivo transplantation experiments into mice and it is not clear to me how the NCAM+ population contributes to a better survival of animals. As rightly pointed out by the authors the p-value ($p=0.037$) NCAM+ vs NCAM- cells did not reach a very high statistical significance. I believe additional experiments need to be performed to allow firm conclusions.

The time course experiment in Fig. 7b suggests that cells can integrate into kidneys, but then get diluted out over time. The authors performed a simple PCR analysis to determine the persistence of human cells in kidneys, but it is not clear to me how quantitative this measure is. Real time PCR would be better suited for quantification and may even allow estimation of the number of human cells present in the kidneys. Also, why are there no values for the experiments with NCAM- cells? Do they integrate less/more into kidneys (comparison between NCAM+ and NCAM- cells)?

Fig7c shows that NCAM+ cells can be found in mouse kidneys, but from the data provided it is not clear to which kidney structure they contribute. Here a much more thorough analysis is required. Do the cells integrate into nephrons? Which differentiation markers do they express (high power views and immunostaining)? Also, I am wondering whether the experiments have been reproduced with NCAM positive/negative cells isolated from distinct donor tissue. I think this is important to corroborate the general conclusion that NCAM positive cells may have beneficial effects in treating renal disease.

Referee #3 (Comments on Novelty/Model System):

This is an interesting report but there are many problems in the current manuscript. There are many RT-PCR data but no immunocytochemistry to confirm protein expression by the cells. There is not sufficient clarity on the statistical significance of the PCR data. The animal studies have to be interpreted as indicating that there is no difference between infusion of NCAM + or NCAM- cells. The saline controls are not relevant here since the effects of cellular infusion is likely to reflect paracrine effects. The LTA, DBA and THP staining of the explants on CAM is very interesting. There are many errors of spelling and usage, some of which we point out in the review.

Referee #3 (Other Remarks):

The authors report the identification of human nephron epithelial progenitor cells in aborted human fetuses. These cells are isolated based on NCAM1 expression and represent a mitotically active population, which when introduced onto the chorio-allantoic membrane of the chick embryo formed proximal tubules, loop of henle, and distal nephron tubules. The authors also injected these cells into mice challenged with glycerol to induce acute kidney injury. The senior author, Dr. Benjamin Dekel, has published a number of papers on the use of NCAM1 as a marker for nephron progenitors in the developing human kidney.

1. The strength of the manuscript lies in the demonstration that the NCAM1+ cell population can give rise to kidney tubular structures in vivo. The characterization of the different cell populations isolated from the human fetal kidneys would be improved by some protein expression analysis (preferably by immunocytochemistry or western blot to show expression and localization of key transcription factors reported to be expressed in these cells). This is an important limitation of the work.

2. The data on glycerol-induced acute renal failure protection is weak. The authors find no statistical difference between the infusion of NCAM negative cells and NCAM positive cells. Clearly it is

most likely that the effects of protection are related to paracrine effects, and those paracrine effects appear to be similar in the NCAM- cells versus NCAM+ cells. There are no data on creatinine.

3. When many of the expression levels are described from experiments using RTPCR, there are percentages presented and apparent differences in some cases which are relatively small and no clear statistical difference is identified, so it is hard to know which of the differences presented, for example in figure 1, are meaningful. The authors tend to select some and ignore others.

4. In figure 1b, the authors characterize the HFK cells in SFM and SCM conditions using RT-PCR. It would be informative for the authors to demonstrate protein expression and localization in SFM and SCM-cultured cells via immunostaining of some of these markers, such as ECAD, vimentin, PAX2, SIX2, and WT1, all of which have very good staining antibodies. This might also help to demonstrate whether these cultures are homogeneous or heterogeneous. Co-staining for ECAD and vimentin could help clarify which cells express these markers, since vimentin is a marker of mesenchymal rather than epithelial cells.

5. In figure 1b and 1c, it is not clear which SFM and SCM populations were used for comparison. Were all experiments done on PO day 7 SCM and PO day 14 SFM cultures? If so, why were day 7 and day 14 chosen, respectively? Is there any change in gene expression profiles within either SCM or SFM cultures over time?
6. In figure 2 d-e, the authors try to characterize single cell clones of the SCM-cultured cells. What passage cells were used for each clone for the analyses? Was there a significant change in gene expression with each passage? Also, there is significant heterogeneity in the gene expression patterns between clone 1 and clone 2 which the authors do not interpret.

7. In Figure 3, much of the characterization of the NCAM1+ SFM cells is done by RT-PCR. The authors should try to immunostain these cells with well-established antibodies against PAX2, SIX2, and WT1 to show protein expression/localization.

8. In the discussion, the authors talk about NCAM1 expression and state on page 27 that no NCAM1 is detected in epithelial derivative in adulthood. It is known, however, and published in experiments in rats that ischemia is associated with an expression of NCAM1.

9. It is not clear on what basis the authors come to the conclusion that the NCAM+ cells represent a heterogeneous mixture of lineage-restricted epithelial progenitors.

10. On page 27, seven lines from the bottom, it is not clear what references 10 and 11 refer to. In addition, in a number of other places in the text, the references are incomplete, with the authors name present but the year not included.

11. There are a number of typos/spelling errors in the text.
12. 1. Page 4, line 1: "capping mesenchyme" should be changed to "cap mesenchyme".
13. 2. Page 5, line 13: "Iscoves's" should be changed to " Iscoves' ". "Mod" should be spelled out "Modified".
14. 3. Under materials and methods, "Establishment of a primary culture from human kidney", the authors state that after the digesting medium is removed, the cells are resuspended in a "growth medium" without any further description. This should be changed to "either serum-containing or serum-free medium".
15. 4. Page 6, line 9: "transferring" should be changed to "transferrin".
16. 5. Page 6, line 10: "sodium selantine" should be changed to "sodium selenite".
17. 6. Page 6, line 25: Do the authors intend "PBSX1" to be "1X PBS"?
18. 7. Page 9, line 4: "magneticly" should be changed to "magnetically".
19. 8. Page 9, line 5: "invitrogen" should be changed to "Invitrogen"
20. 9. Page 9, line 15: "algoritm" should be changed to "algorithm".
21. 10. Page 9, line 19: the comma after "cluster analysis" should be removed.
22. Page 18, line 16: "capping mesenchyme" should be changed to "cap mesenchyme".
23. Page 18, line 16: "C-" should be changed to "comma-" for clarification.

24. In figure 3b, the style of error bars used for the PAX2 panel are different from the error bars in all the other panels. The authors should try to maintain consistency of style within the figure.
25. Page 19, line 15: "derivates" should be changed to "derivatives".
26. In figure 3b, the Y-axis on the SALL1 panel appears to be mislabeled. The Y-axis reads from bottom to top, "0, 1, 1, 2, 2".

1st Revision - authors' response 17 January 2013

Thank you for your letter from June 6th 2012 and the opportunity to submit a revised manuscript. We wish to thanks the referees for their excellent comments. We have now performed lengthy experiments and extensively revised our manuscript in accordance with the reviewer's suggestions. As we emphasize it is well accepted that one cannot derive genuine renal progenitors from the most accessible stem cells located in the blood or marrow.
Our paper demonstrates for the first time how human tissue, the fetal kidney, can be used to derive nephron progenitor cells that can expanded in culture, generate renal structures and have the ability to halt progression of chronic kidney disease in mice. Ultimately, this comprehensive study affords a bridge from the bench to bedside for the benefit of medicine.
I hope you will find the manuscript suitable for EMBO Molecular Medicine

A point to point address of refs' comments is listed below:

Referee #1 (Comments on Novelty/Model System):

The mouse is a tractable and affordable model in which to test therapeutic engraftment of human tissue. For translational purposes, testing in additional models will be required, but for the proof of principle experiment described in this paper a rodent model is adequate.

Thank you for this comment. Importantly, we have extended our translational analysis of human nephron precursors to include a chronic kidney disease mouse model in addition to the acute one and show in proof-of -principle experiments the ability of human nephron progenitor cells to halt progression of chronic kidney disease (new Fig. 8 and new Supp Fig. 8).

Referee #1 (Other Remarks)

The authors have generated an isolation and culture system for nephron progenitor cells from embryonic human kidney tissue. They have used this method to derive kidney cells displaying gene expression characteristics of nephron progenitors, and have subsequently engrafted these cells into two model systems: i) Chick chorioallantoic membrane, ii) Mice with glycerol induced acute kidney injury. They find that the progenitor cells form epithelial structures in the chick assay, and that there is some protective effect of engraftment in the mouse model of kidney injury. Alternative sources of kidney tissue is an active area of research because of the clinical need, and the work is potentially significant. Although the authors have conducted a thorough and convincing analysis of the purified cells, the novelty and impact of the paper rest on the finding that injected cells are renoprotective in a model of acute kidney injury, which is the weakest experiment in the manuscript. Several issues surrounding the interpretation of results and choice of model need to be clarified to substantiate the authors' conclusions:

1. There is no lineage analysis (human vs chick) in the chick experiment showing the origin of the epithelial structures that arise following engraftment. This is a standard proof for an engraftment experiment and it should be included.

Thank you for this comment. We have immunostained the chick CAM with human specific markers that do not cross react with chick tissue and show human - specific labeling of epithelial structures. These data have been added to supplementary (new Supp Fig. 7)

2. The authors do not show engraftment of injected cells into the kidney, they simply show that Qdot labeled cells localize to the kidney. Trapping of cells and possibly free Qdots in the fine vasculature of the kidney is anticipated, so simply showing a low magnification shot of Qdot signal in the organ

is not convincing - the authors need to evaluate histologically if Qdot labeled cells are incorporated into kidney structures to see if there is tissue engraftment, or if Qdots and cells are merely trapped. A control showing that Qdot-loaded particles the size of a cell, or unrelated cells loaded with Qdots, do not stick in the kidney would be a welcome addition to this dataset.

Thank you for this comment. The acute kidney model evaluates a therapeutic effect 72hr after its induction. We observed a significant reduction (indeed not highly significant) in the proportion of mice that develop acute renal failure following injection of hNPCs. We show by mCherry labeling cells and by a tracing human mRNA using calibrated human specific real-time PCR which allows identification of a small amount of human cells that hNPCs are indeed engrafted in the kidney. Nevertheless, we believe that 72hr does not allow for generation of kidney structures (differentiation to nephron epithelial cells) and such early effects are via paracrine mechanisms as observed for other cell types such as BM-MSCs (see ref's comment below). Moreover, we have added new data of human specific gene expression in the murine lungs showing that following IV injection the strongest signal is in the lung (hGAPDH qRT-PCR: lungs : C_t=20, kidneys: C_t=30). This indicates that the IV route is not ideal for renal engraftment and may limit the functional effects of hNPCs in this injury model.

To address the *in vivo* part and strengthen the translational relevance we have now added lengthy experiments (14w) employing the 5/6 nephrectomy model. This is a model that simulates chronic progressive renal injury. Following our observation that human fetal NCAM+ cells harbor clonogenic and progenitor properties and are unique in their ability to generate human epithelial structures in the CAM, we have evaluated their ability to influence renal function (creatinine clearance, CrCl) in the 5/6 nephrectomy model. We now show for the first time that repeated hNPC injection leads to significant therapeutic effects in this stringent model. These functional results can be linked to engraftment, differentiation and integration potential of hNPCs in the diseased murine kidneys, suggesting that lineage differentiation is likely to contribute, at least in part, to the therapeutic effects (See new Fig. 8 and Supp Fig. 8).

3. Some of the earliest experiments done to assess the renoprotective effects of stem cells were confounded by the fact that the cells produce renoprotective factors that are secreted into the culture medium. Thus, removing the cells from the engraftment mixture did not significantly reduce the renoprotective effect of the "engraftment". Lloyd Cantley's group has published several studies on this phenomenon, which may be very significant clinically. To support the last sentence of the abstract, it would be important to know whether the NCAM+ cells are exerting their renoprotective effect through cellular engraftment or production of soluble renoprotective factors.

Thank you for this comment. Importantly, the ATN glycerol model results mostly in renal dysfunction 48- 72 hrs after glycerol injection and the beneficial effects observed at this timepoint following early cell administration (IV, 2h after glycerol administration) are therefore likely to be paracrine dependent. Histological analysis would be also less relevant here. We have extended our observations on renal function improvement and repeated hNPC administration to the prolonged chronic kidney model for which we could clearly show cellular engraftment (new Fig. 8 and new Supp Fig. 8)

4. The choice of injury model is confusing because the ATN following glycerol injection is secondary to muscle injury. No clinical chemistry is provided to prove that the cell injection is not protecting the mice from rhabdomyolysis, with a secondary ameliorative effect on kidney injury.

Thank you for this important comment. The ATN model we have employed is well described for studying the effects of cell therapy and has been used by other groups (Sagrinati et al, 2006). Nevertheless, to ascertain that we are not model-dependent we have now performed additional *in vivo* experiments in an independent chronic injury model (new Fig. 8 and new Supp Fig. 8).

Additional points:

qPCR is not be the best way to tell if cells in the NCAM fraction are enriched for epithelial progenitors. Six2 is likely expressed by other cells at lower levels and maybe these are eliminated in the NCAM positive fraction. SIX2 co-stain on the NCAM fraction would be conclusive.

Thank you for this comment. We have added immunostaining of SIX2 in the NCAM+ fraction and show prominent staining (See new Fig 2). Importantly, not all NCAM+ cells co-stain for the SIX2 marker indicating – as we discuss in the manuscript – that NCAM+ fraction also contains early and more committed nephron epithelial precursors beyond the SIX2 stage (including early nephron derivatives of the SIX2 population such as S – shaped bodies). Importantly, NCAM+ cells show reduced expression of E-CAD and mildly elevated vimentin staining compared to NCAM- cells (corroborating with qRT-PCR data) indicating that hNPCs have not completed the MET process towards mature nephron epithelia and can be considered as differentiating progenitors (new Fig. 2).

DBA lectin is an odd choice to mark distal tubule as it is generally used to mark collecting duct quite specifically in the mouse (Laitinen et al. 1987. Changes in the glycosylation pattern during embryonic development of mouse kidney as revealed with lectin conjugates. J HistochemCytochem 35:55-65). If DBA marks distal tubule in the human kidney, the authors should provide a reference. If not, it would mean that structures of collecting duct identity develop from the NCAM+ fraction, which would either indicate contamination of the cell preparation used for engraftment, or that cells of the nephron lineage have transdifferentiated to the collecting duct lineage in this assay.

Thank you for this comment. As shown in (Murata et al, 1983) DBA can also be observed in distal tubules.

It seems bold to say in the abstract that human nephron progenitor cells prevent death and renal failure when all that was observed was a trend. If the authors want to make this claim they need to analyze an appropriate number of mice based on a power analysis of their current data.

Thank you for this comment. Our analysis shows that the proportion of mice developing acute renal failure is significantly lower when injecting human NCAM+ cells (Fisher Exact test and chi square analysis). We have toned down our claims in the abstract. In the 5/6 nephrectomy model we have added new data showing at 12 weeks significant improvement in renal function –CrCl compared to baseline CrCl and that a significant lower amount of mice deteriorate renal function when receiving repeated injections of hNPCs (new Fig. 8 and new Supp Fig. 8).

Referee #2 (Other Remarks)

This manuscript describes the isolation of a population of cells from human fetal kidneys that can be expanded in vitro, have characteristics of nephron progenitor cells and when transplanted on chick chorioallantoic membrane can give rise to structures that express nephron specific markers. Moreover, the authors performed experiments in which transplantation of NCAM+ cells provide some protective effect from glycerol induced acute renal failure.
The isolation and identification of a human nephron progenitor cells is an important goal, as it may in the long run help treating renal diseases. The manuscript by Metsuyanim et al., provides some important steps towards achieving this goal. Unfortunately, several experiments are incomplete and a number of issues need to be resolved before I can support publication.

1 .The first part of the paper describes the culture and characterization of minced human embryonic kidney in serum free and containing medium. Figure 1 shows quantitative analysis of several genes expressed within the developing kidney, but I am wondering how strong the expression is, when compared to wildtype developing kidneys/cap mesenchyme. Also, I don't understand why no error bars are shown for SCM samples. I realize that the value for the SCM cultures was set to one, but if 3 independent samples have been used (figure legends), I would expect to have some variability between the individual samples.

Thank you for the comment. a. A comparison of cells to tissues is likely t be irrelevant here. We repeatedly observe that when comparing human fetal kidney cells to fragments of human kidney tissue there is global dampening of gene expression. We could detect this when performing microarrays comparing hFK tissue and hFK cells. b. Comparative real time RT-PCR analysis of gene expression were analyzed using SDS RQ Manager 1.2 software or StepOne Software v2.1 using the well accepted method of ΔΔCT such that the expression levels of the calibrator samples, in

this case SCM samples =1, therefore in each biological repeat irrespective of the absolute expression level, the calibrator is =1.

2. Limited diluted cells expanded in SCM form clones, but appear to undergo a crisis at about passage 5, a process that is often observed in primary cultures. In the following analysis only 2 independent clones appeared to be analysed. Since the variation of expression of these two clones is quite substantial, I am not sure one can draw clear-cut conclusions. This is even more worrying given substantial variability of gene expression in clone 1 and 2 for several of the genes analysed (CD90, CD105, Foxd1, Sall1, vimentin...). I realize that human material is limited, but given the low sample
number, I am somewhat concerned regarding the reproducibility of the data?

Thank you for this comment. A means for isolating kidney stem cells from human fetal kidneys would be limiting dilution analysis followed by clonal expansion indicative of self-renewal. Importantly, we wanted to show that clonal self –renewing populations can be isolated from the human fetal kidney in the adhesion cultures supplemented with serum. However, these long-term clonal expansions appear as mesenchymal stem-like cells (morphology and gene/protein expression) and not nephron epithelial progenitors which can be expanded to some degree in serum free conditions. A recent paper (Lusis et al, Stem Cell Res) showed nephrospheres as means to enrich embryonic nephron progenitors. Nevertheless, nephrospheres just like our long-term MSC-like clonal populations fail to epithelialize suggesting these methods are not optimal for derivation of cells suitable for nephron formation, e.g. kidney-forming cells. We acknowledge that only a limited number of self-renewing clones can be expanded from human fetal kidneys and hence the limited data on two clones. However, we believe that these data should be included as it emphasizes the relevance of SFM media expansion and NCAM immunosorting for derivation of committed nephron progenitors that generate epithelial structures (harbor direct kidney-forming capacities). We have re-wrote this paragraph as to make it more clear for the reader and moved results to Supplementary (Supp Fig. 1).

3. Transplantation experiments into the chick chorioallantoic membrane suggest that NCAM+ cells can develop into epithelial structure that express markers of various nephron segments and the authors conclude that the transplanted cells have undergone differentiation into nephrons. However, NCAM positive cells are not restricted to the cap mesenchyme (progenitor compartment), but can also be found within epithelial comma- and S-shaped bodies (Supp. Fig1). It is not clear to me whether the epithelial cells detected after transplantation are derived from mesenchymal cells (differentiation) or whether they represent epithelial cells already present in the original NCAM+ population (and maybe reassembled as tubules).***

Thank you for this comment. As the referee correctly points out and as we show in Supp fig. 4, NCAM expression in human fetal kidney is in the CM but also in early nephron derivatives. When we stain human NCAM+ cells for Six2 there exist a double positive population but also more differentiated NCAM+ cells that lack the Six2 marker. After grafting onto the chick CAM and the 5/6 Nx model and the generation of renal tubular structures, cells express epithelial markers and importantly lose NCAM1 expression indicating maturation (New Fig. 7 Fig. 8, Supp Fig. 7 and Supp Fig. 8). Moreover, single cells that have engrafted *in vivo* but have yet differentiated into renal tubules are negative for epithelial markers (panCK/MNF116 that we have used). They retain MNF116 expression once differentiated into tubule-structure.

4. My biggest problem with this paper relates to the in vivo transplantation experiments into mice and it is not clear to me how the NCAM+ population contributes to a better survival of animals. As rightly pointed out by the authors the p-value (p=0.037) NCAM+ vs NCAM- cells did not reach a very high statistical significance. I believe additional experiments need to be performed to allow firm conclusions.

Thank you for this comment. The acute kidney model evaluates a therapeutic effect 72hr after its induction. We observed a significant reduction (indeed not highly significant) in the proportion of mice that develop acute renal failure following injection of hNPCs. We show by mCherry labeling and by a tracing human mRNA using calibrated human specific real-time PCR which allows identification of a small amount of human cellsthat hNPCs are indeed engrafted in the kidney. Nevertheless, we believe that 72hr does not allow for generation of kidney structures (differentiation

to nephron epithelial cells) and such early effects are via paracrine mechanisms as observed for other cell types such as rBM-MSCs (see ref's comment below). Moreover, we have added new data of human specific gene expression in the murine lungs showing that following IV injection the strongest signal is in the lung (hGAPDH qRT-PCR: lungs : C_t=20, kidneys: C_t=30). This indicates that the IV route is not ideal for renal engraftment and may limit the functional effects of hNPCs in this injury model.

To more adequately address the *in vivo* part and strengthen the translational relevance we have now added lengthy experiments (14w) employing the 5/6 nephrectomy model. This is a model that simulates chronic progressive renal injury. Following our observation that human fetal NCAM+ cells harbor clonogenic and progenitor properties and are unique in their ability to generate human epithelial structures in the CAM, we have evaluated their ability to influence renal function (creatinine clearance, CrCl) in the 5/6 nephrectomy model. We now show for the first time that repeated hNPC injection leads to significant therapeutic effects in this model (For testing a therapeutic effect we employed a protocol with three direct injections of hNPCs over a 14 week time period). We found a clear separation between treated and non-treated mice after the third injection; at 12 weeks we observed significant improvement in renal function –CrCl compared to baseline CrCl and that a significant lower amount of mice deteriorated renal function when receiving repeated injections of hNPCs. We believe that these set of chronic experiments substantiate our translational findings in the acute model and provide proof of principle experiments towards clinical implementation for which the greatest need exists (CKD) (new Fig. 8 and new Supp Fig. 8).

The time course experiment in Fig. 7b suggests that cells can integrate into kidneys, but then et diluted out over time. The authors performed a simple PCR analysis to determine the persistence of human cells in kidneys, but it is not clear to me how quantitative this measure is. Real time PCR would be better suited for quantification and may even allow estimation of the number of human cells present in the kidneys. Also, why are there no values for the experiments with NCAM- cells? Do they integrate less/more into kidneys (comparison between NCAM+ and NCAM- cells)?

Thank you for this comment. Importantly, the ATN glycerol model results mostly in renal dysfunction 48- 72 hrs after glycerol injection and the beneficial effects observed at this timepoint following early cell administration (IV, 2h after glycerol administration) are therefore likely to be paracrine dependent. We indeed present real-time and not "simple" PCR data (the real-time PCR product was analyzed by ethidium agarose gel and according to your comment we mention Ct values). These results were further complemented by mCherry cell labeling and tracking (new Fig. 7). The IV route results in major lung engraftment and less so kidney engraftment (see Ct values) hampering functional effects of hNPCs. Histological analysis would be also less relevant here so is lineage differentiation and in fact many other cell types have the capacity to function here in a paracrine matter (this has been widely shown for MSCs). Following the evidence accumulated by *in vitro* assays and the chick CAM *in vivo* assay indicating human fetal NCAM1+ cells grown in SFM to fulfill hNPC criteria we wanted to show here that hNPCs have this paracrine capacity to influence acute disease like other cell types but are definitely not unique in that sense and this is not the major translational focus. We emphasize that in the text. We have extended our observations on renal function improvement and repeated hNPC administration to the prolonged chronic kidney model for which we could clearly show cellular engraftment and integration (new Fig. 8 new Supp Fig. 8)

Fig7c shows that NCAM+ cells can be found in mouse kidneys, but from the data provided it is not clear to which kidney structure they contribute. Here a much more thorough analysis is required. Do the cells integrate into nephrons? Which differentiation markers do they express (high power views and immunostaining)? Also, I am wondering whether the experiments have been reproduced with NCAM positive/negative cells isolated from distinct donor tissue. I think this is important to corroborate the general conclusion that NCAM positive cells may have beneficial effects in treating renal disease.

Thank you for this comment. We employed the 5/6 nephrectomy model to evaluate these parameters and show engraftment, de-novo differentiation/epithelial structure formation and integration of hNPCs in chronically diseased kidneys (See new Fig. 8, supp Fig. 8).

Referee #3 (Comments on Novelty/Model System):

This is an interesting report but there are many problems in the current manuscript. There are many RT-PCR data but no immunocytochemistry to confirm protein expression by the cells. There is not sufficient clarity on the statistical significance of the PCR data. The animal studies have to be interpreted as indicating that there is no difference between infusion of NCAM + or NCAM- cells. The saline controls are not relevant here since the effects of cellular infusion is likely to reflect paracrine effects. The LTA, DBA and THP staining of the explants on CAM is very interesting. There are many errors of spelling and usage, some of which we point out in the review.

Referee #3 (Other Remarks):

The authors report the identification of human nephron epithelial progenitor cells in aborted human fetuses. These cells are isolated based on NCAM1 expression and represent a mitotically active population, which when introduced onto the chorio-allantoic membrane of the chick embryo formed proximal tubules, loop of henle, and distal nephron tubules. The authors also injected these cells into mice challenged with glycerol to induce acute kidney injury. The senior author, Dr. Benjamin Dekel, has published a number of papers on the use of NCAM1 as a marker for nephron progenitors in the developing human kidney.

1. The strength of the manuscript lies in the demonstration that the NCAM1+ cell population can give rise to kidney tubular structures in vivo. The characterization of the different cell populations isolated from the human fetal kidneys would be improved by some protein expression analysis (preferably by immunocytochemistry or western blot to show expression and localization of key transcription factors reported to be expressed in these cells). This is an important limitation of the work.

Thank you for this comment. We have added immunostaining of Six2 in the NCAM+ fraction and show prominent staining (See new Fig 2). Importantly, not all NCAM+ cells co-stain for the Six2 marker indicating – as we discuss in the manuscript – that NCAM+ fraction also contains early and more committed nephron epithelial precursors beyond the Six2 stage (including early nephron derivatives of the SIX2 population such as S – shaped bodies). Importantly, NCAM+ cells show reduced expression of E-CAD compared to NCAM- cells indicating that they have not completed the MET process towards mature nephron epithelia and can be considered as differentiating progenitors (See new Fig 2).

2. The data on glycerol-induced acute renal failure protection is weak. The authors find no statistical difference between the infusion of NCAM negative cells and NCAM positive cells. Clearly it is most likely that the effects of protection are related to paracrine effects, and those paracrine effects appear to be similar in the NCAM- cells versus NCAM+ cells. There are no data on creatinine.

Thank you for this comment. We totally agree with the referee. The acute kidney model evaluates a therapeutic effect 72hr after its induction. We show by mCherry labeling and by a tracing human mRNA using calibrated human specific real-time PCR which allows identification of a small amount of human cells that hNPCs are indeed engrafted in the kidney. In addition, we observed a significant reduction (indeed not highly significant) in the proportion of mice that develop acute renal failure following injection of hNPCs when compared to saline. Nevertheless, we believe that 72hr does not allow for generation of kidney structures (differentiation to nephron epithelial cells) and such early functional effects are via paracrine mechanisms as observed for other cell types such as BM-MSCs and may well be achieved by varying cell types. Moreover, we have added new data of human specific gene expression in the murine lungs showing that following IV injection the strongest signal is in the lung (hGAPDH qRT-PCR: lungs : Ct=20, kidneys: Ct=30). This indicates that the IV route is not ideal for renal engraftment and may limit the functional effects of hNPCs in this injury model (accordingly, creatinine levels were indeed less affected than urea levels). To more adequately address the *in vivo* part and strengthen the translational relevance we have now added lengthy experiments (14w) employing the 5/6 nephrectomy model. This is a model that simulates chronic progressive renal injury. Following our observation that human fetal NCAM+ cells harbor clonogenic and progenitor properties and are unique in their ability to generate human epithelial structures in the CAM, we have evaluated their ability to influence renal function (creatinine

clearance, CrCl; the most stringent biochemical measurement) in the 5/6 nephrectomy model. We now show for the first time that repeated hNPC injection leads to significant therapeutic effects in this model (For testing a therapeutic effect we employed a protocol with three direct injections of hNPCs over a 14 week time period). We found a clear separation between treated and non-treated mice after the third injection; at 12 weeks we observed significant improvement in renal function – CrCl compared to baseline CrCl and that a significant lower amount of mice deteriorated renal function when receiving repeated injections of hNPCs. We believe that these set of chronic experiments substantiate our translational findings in the acute model and provide proof of principle experiments towards clinical implementation for which the greatest need exists (CKD) (new Fig. 8)

3 .When many of the expression levels are described from experiments using RTPCR, there are percentages presented and apparent differences in some cases which are relatively small and no clear statistical difference is identified, so it is hard to know which of the differences presented, for example in figure 1, are meaningful. The authors tend to select some and ignore others.

We have re-wrote this section according to the ref's comment and addressed meaningful data in the text.

4. In figure 1b, the authors characterize the HFK cells in SFM and SCM conditions using RT-PCR. It would be informative for the authors to demonstrate protein expression and localization in SFM and SCM-cultured cells via immunostaining of some of these markers, such as ECAD, vimentin, PAX2, SIX2, and WT1, all of which have very good staining antibodies. This might also help to demonstrate whether these cultures are homogeneous or heterogeneous. Co-staining for ECAD and vimentin could help clarify which cells express these markers, since vimentin is a marker of mesenchymal rather than epithelial cells.

Thanks for this comment. Pax2 is not overexpressed in NCAM+ cells as it strongly expressed in UB/early UB derivatives which are NCAM-. We stained Six2, vimentin and E-cadherin and demonstrate that a fraction of NCAM+ cells, which shows reduced E-CAD staining, expresses Six2 but not in all cells indicating early and more differentiated epithelial progenitors within the NCAM+ fraction.

5. In figure 1b and 1c, it is not clear which SFM and SCM populations were used for comparison. Were all experiments done on PO day 7 SCM and PO day 14 SFM cultures? If so, why were day 7 and day 14 chosen, respectively? Is there any change in gene expression profiles within either SCM or SFM cultures over time?

Thanks for this comment. Both cell cultures start at similar concentrations. However, in SFM, cells adhere less are smaller and therefore reaching confluence in P0 is longer. This is the reason for different timepoints we show in Fig. 1. As we mention in the text from P1 and thereafter doubling time is similar.

6. In figure 2 d-e, the authors try to characterize single cell clones of the SCM-cultured cells. What passage cells were used for each clone for the analyses? Was there a significant change in gene expression with each passage? Also, there is significant heterogeneity in the gene expression patterns between clone 1 and clone 2 which the authors do not interpret.

Thank you for this comment. A means for isolating kidney stem cells from human fetal kidneys would be limiting dilution analysis followed by clonal expansion indicative of self-renewal. Importantly, we wanted to show that clonal self –renewing populations can be isolated from the human fetal kidney in the adhesion cultures supplemented with serum. However, these long-term clonal expansions appear as mesenchymal stem-like cells (morphology and gene/protein expression) and not nephron epithelial progenitors which can be expanded to some degree in serum free conditions. A recent paper (Lusis et al, Stem Cell Res) showed nephrospheres as means to enrich embryonic nephron progenitors. Nevertheless, nephrospheres just like our long-term MSC-like clonal populations fail to epithelialize suggesting these methods are not optimal for derivation of cells suitable for nephron formation, e.g. kidney-forming cells. We acknowledge that only a limited number of self-renewing clones can be expanded from human fetal kidneys and hence the limited data on two clones. However, we believe that these data should be included as it emphasizes the

relevance of SFM media expansion and NCAM immunosorting for derivation of committed nephron progenitors that generate epithelial structures (harbor direct kidney-forming capacities). We have re-wrote this paragraph as to make it more clear for the reader and moved results to Supplementary (Supp Fig. 1).

7. In Figure 3, much of the characterization of the NCAM1+ SFM cells is done by RT-PCR. The authors should try to immunostain these cells with well-established antibodies against PAX2, SIX2, and WT1 to show protein expression/localization.

Thanks for this important comment. We have addressed the ref's concern in comment 1.

8. In the discussion, the authors talk about NCAM1 expression and state on page 27 that no NCAM1 is detected in epithelial derivative in adulthood. It is known, however, and published in experiments in rats that ischemia is associated with an expression of NCAM1.

We have now indicated this and added reference

9. It is not clear on what basis the authors come to the conclusion that the NCAM+ cells represent a heterogeneous mixture of lineage-restricted epithelial progenitors.

We have now with Six2 staining show the NCAM+ fraction contain earlier and more committed nephron progenitors. We have revised this part of the discussion so as to make the comment more clear.

10. On page 27, seven lines from the bottom, it is not clear what references 10 and 11 refer to. In addition, in a number of other places in the text, the references are incomplete, with the authors name present but the year not included.

.11*There are a number of typos/spelling errors in the text.*
.1 .12*Page 4, line 1: "capping mesenchyme" should be changed to "cap mesenchyme."*
.2 .13*Page 5, line 13: "Iscoves's" should be changed to " Iscoves' ". "Mod" should be spelled out "Modified."*
.3 .14*Under materials and methods, "Establishment of a primary culture from human kidney", the authors state that after the digesting medium is removed, the cells are resuspended in a "growth medium" without any further description. This should be changed to "either serum-containing or serum-free medium."*
.4 .15*Page 6, line 9: "transferring" should be changed to "transferrin."*
.5 .16*Page 6, line 10: "sodium selantine" should be changed to "sodium selenite."*
.6 .17*Page 6, line 25: Do the authors intend "PBSX1" to be "1X PBS?"*
.7 .18*Page 9, line 4: "magneticly" should be changed to "magnetically."*
.8 .19*Page 9, line 5: "invitrogen" should be changed to "Invitrogen"*
.9 .20*Page 9, line 15: "algoritm" should be changed to "algorithm."*
.10 .21*Page 9, line 19: the comma after "cluster analysis" should be removed.*
.22*Page 18, line 16: "capping mesenchyme" should be changed to "cap mesenchyme."*
.23*Page 18, line 16: "C-" should be changed to "comma-" for clarification.*
.24*In figure 3b, the style of error bars used for the PAX2 panel are different from the error bars in all the other panels. The authors should try to maintain consistency of style within the figure.*
.25*Page 19, line 15: "derivates" should be changed to "derivatives."*
.26*In figure 3b, the Y-axis on the SALL1 panel appears to be mislabeled. The Y-axis reads from bottom to top, "0, 1, 1, 2, 2."*

Thank you for these detailed and important comments.
COMMENTS 10, 11 (1-25) BELOW HAVE ALL BEEN ADDRESSED.

2nd Editorial Decision 05 March 2013

Thank you for the submission of your manuscript to EMBO Molecular Medicine. We have now heard back from the three referees whom we asked to evaluate your manuscript. As you will see

from the reports below, the referees appreciate the provision of a new model but still raise substantial concerns on your work.

Referee 1 is the most positive but suggests to considerably shorten your paper, remove data and consolidate the message in a short format.

Referees 2 and 3 raise concerns regarding the provided new experiments (SIX2 labeling to improve in figure 2 and costaining to use in figure 8 immunofluorescence data to increase conclusiveness). Referee 1 still questions statistical meaning and referee 3 requires protein expression evidence, both issues were already asked to be addressed in my previous letter.

As you know, we would normally not allow a second revision. I am prepared in this case, however, to give you another opportunity to improve your manuscript and respond convincingly to all issues raised, with the understanding that acceptance or rejection of the manuscript will depend on the satisfaction of the referees in the final version of the manuscript. We agree with referee 1 and would like to kindly encourage you to revise the paper as a short report with a maximum of 4 figures (please see our Guide to Authors for help in formatting).

As you know, EMBO Molecular Medicine has a "scooping protection" policy, whereby similar findings that are published by others during review or revision are not a criterion for rejection. However, I do ask you to get in touch with us after three months if you have not completed your revision, to update us on the status. Please also contact us as soon as possible if similar work is published elsewhere.

I look forward to seeing a revised form of your manuscript as soon as possible.

***** Reviewer's comments *****

Referee #1 (Remarks):

In this revision of their manuscript Metsuyanim et al. have made a great effort to address the reviewers' comments, including several new experiments. Most importantly, a new and more convincing mouse disease model has been included. Overall, although the authors provide an interesting dataset the work needs linguistic revision and abbreviation - there are typos and stray punctuation marks, and the results and discussion sections would benefit from editing to improve the clarity and flow. In all, the text could be much more concise.

These points need to be addressed:

Several of the graphs are lacking error bars and it would be helpful to include the statistical significance on the graphs (eg Figs 3, 4, 7).

Some of the bar graphs have error bars that seem too large for reliable interpretation of the data (eg Fig 3E).

Figs 1-4 should be consolidated into one or 2 figures. The current volume of individual panels and bar graphs is daunting for the reader and not proportional to the point the authors are making. As an example, couldn't the authors find a way to condense all of the individual panels in 1b into a single small bar graph?

Fig 5: panels a and b don't add anything - the authors should omit them and replace with a schematic outlining the experimental plan.

Fig 7: Considering all of the caveats around interpretation of this experiment I don't think it should be included in the paper at all. In my opinion the paper would be much stronger without this data - it doesn't provide any answers and generates a lot of questions that can only be answered by speculative arguments.

Fig 9: The message of this figure can be expressed in a single sentence. It does not add anything and should be removed.

Referee #2 (Comments on Novelty/Model System):

The isolation of human nephron progenitors using NCAM as a marker for FACS sorting is interesting, but some of the data are of low quality and could be improved (see comments below). The fact that renal progenitor cells can differentiate into nephron cells is per se not too surprising.

Referee #2 (Remarks):

The present manuscript is a much improved version over the initial submission and the authors have performed added additional experiments most notably employing a second renal disease model (5/6 nephrectomy).

Listed below are still some points that should be addressed.

Response of authors to my original comment 1:

Comparative real time RT-PCR analysis of gene expression were analyzed using SDS RQ Manager 1.2 software or StepOne Software v2.1 using the well accepted method of ΔΔCT such that the expression levels of the calibrator samples, in this case SCM samples =1, therefore in each biological repeat irrespective of the absolute expression level, the calibrator is =1.

While I am not familiar with this particular software, I respectfully disagree with the authors' response. The method used allows comparison between one sample under different conditions (in this case SFM and SCM). However, I was interested in the variation between samples isolated from different kidneys. Biological samples are bound to show a certain degree of variation in gene expression and setting the 'control' sample to 1 hides this variability.

Figure 2:
Images for immunostainings in Figure 2 C and D are of too low resolution. High power views should to be provided.
The staining for SIX2 is worrisome. SIX2 is a transcription factor and should be almost exclusively within the nucleus. The images and video provided does not give me this impression. Either the staining has not worked probably (what do control kidney samples look like?), or the authors only detect background staining.
Incidentally, the video (confocal Z-stack) does not provide important information and should be removed.

Figure 6:
The immunostaining data for DBA, LTA and Tamm-horsfall are appreciated, although I would have preferred to see these data in co-staining with a human-specific antigen to ensure that these cells are indeed derived from the transplanted nephron progenitors.

The new experiments on the 5/6Nx chronic renal disease mouse model are highly appreciated and show that human renal progenitor cells can integrate into the diseased kidney and form epithelial structures. Unfortunately, images 8j-l are difficult to interpret and it is not clear whether cells are truly integrating into damaged tubules or whether they form independent tubules that are not hooked up to the general collecting system. Higher power images using costaining of HLA and E-cadherin or HLA and tubule segment specific markers would be helpful.

Referee #3 (Comments on Novelty/Model System):

I believe there continue to be many problems in the current manuscript. There are many RT-PCR data but insufficient immunocytochemistry to confirm protein expression by the cells. The use of saline controls rather than NCAM- controls in the animal studies remains a significant problem. I have a number of additional concerns.

Referee #3 (Remarks):

The authors have responded adequately to some of my concerns. However there remain a number of issues:

1. Page 5: cells are stated to have doubling time of 32.95 hr. I find it difficult to see numbers like this with no statistical variation presented and with 2 decimal points. This is not the only place this level of precision is implied which is not realistic biologically.

2. Page 5: it is implied that growth in serum results in EMT because there is more fibroblast outgrowth by 7 days. This might rather just as well be related to selection of the cells from the very beginning of the isolation from human fetal kidney.

3. The data on Figure 1c indicate very high expression patterns of proteins. It would be much more reassuring to see immunocytochemistry to demonstrate these staining patterns.

4. Supplemental Figure 2 is not well explained in the legend nor in the text. The figure is also difficult to read because the legends are not printed at a resolution that would be necessary.

5. Figure 6. Is there any staining with any of the antibodies or lectins after NCAM- cell injections? The text on page 11 is not clear on this and there are no data presented.

6. Figures 7a and d should include error bars.
7. Why was human GAPDH used for demonstration of engraftment in the glycerol experiments and b2-microglobulin in the chronic kidney disease experiments? The authors should demonstrate human GAPDH in the chronic experiments. Furthermore, how many animals are reflected in the data presented in figure 8e?

8. The authors have chosen selective measures of efficacy in the animal studies. For example there is a statistical difference in the number of animals who suffered a >15% reduction in creatinine clearance if the NCAM+ cells are injected. This reviewer would like to see the time course of creatinine clearance values for each group as I suspect the differences might be small. Why was 15% chosen? This is not standard.

9. Figure 8h-o: How often quantitatively can the authors conclude that the hNPCs integrate into existing tubules and form new tubules by human specific marker staining? Is it not possible that some of the isolated cell staining may be related to cell death of the injected cells and uptake by other cells of the labeled debris? Also what is seen when NCAM- cells are injected? It is not appropriate to use saline as a control for these experiments without also testing NCAM- cells.

10. The abstract conclusion that these findings have "major potential for cell-based therapeutic strategies" ignores the potential immunological difficulties and the overall difficult ethical issues with deriving cells from human fetuses.

2nd Revision - authors' response 06 June 2013

Editor comments:

Referee 1 is the most positive but suggests to considerably shorten your paper, remove data and consolidate the message in a short format.

Thanks for the encouragement. Accordingly, we have substantially shortened the manuscript and consolidated data into four figures (other data has been removed in according with refs' suggestion or integrated in supplementary figures).

Referees 2 and 3 raise concerns regarding the provided new experiments (SIX2 labeling to improve in figure 2 and costaining to use in figure 8 immunofluorescence data to increase conclusiveness).

Referee 1 still questions statistical meaning and referee 3 requires protein expression evidence, both issues were already asked to be addressed in my previous letter.

Importantly, analysis of protein expression in human cells after sorting to delineate Ncam1/Six2 relationship has proven laborious. Nevertheless, we have performed a series of new IF experiments showing that human Ncam1+ nephron progenitors are not entirely Six2+ but clearly contain the Six2+ population while showing reduced expression of E-cad compared to Ncam1- counterparts (New fig. 2c,d,e,f and new Supp Fig. 4). Moreover to get a detailed look of spatial relationships between Ncam1, Six2 and E-cad we have studied expression in cultured mouse embryonic kidneys. These data clearly show an overlap between Six2 and Ncam in the cap mesenchyme cells and that Ncam1 expresses beyond the Six2 domain in early nephron figures (new Fig. 1a). Indeed, cultured human Ncam1+ cells also contained an Ncam1+Six2- cell population altogether indicating that $Ncam1^{+}$ cells likely was heterogeneous, comprising a mix of stem and more committed progenitor cells.
In accordance with refs suggestion for more protein analysis we have performed new experiments showing in addition to FACS, staining of CD24 and EpCAM in the hFK SFM cell culture (new Supp Fig. 3).
In accordance with ref's suggestions we have now analysed by immunostaining the types of renal structures generated in diseased kidneys and present a more detailed analysis (new Fig. 4, new Supp Fig. 7).
In accordance with ref 1 suggestion, all new figures contain statistical analysis and analyzed for statistical meaning.

As you know, we would normally not allow a second revision. I am prepared in this case, however, to give you another opportunity to improve your manuscript and respond convincingly to all issues raised, with the understanding that acceptance or rejection of the manuscript will depend on the satisfaction of the referees in the final version of the manuscript. We agree with referee 1 and would like to kindly encourage you to revise the paper as a short report with a maximum of 4 figures (please see our Guide to Authors for help in formatting).

In accordance with your insightful suggestion we have generated a revised version along a Report format.
A point to point answer to ref's concerns is presented below;

Referee #1 (Remarks):

In this revision of their manuscript Metsuyanim et al. have made a great effort to address the reviewers' comments, including several new experiments. Most importantly, a new and more convincing mouse disease model has been included. Overall, although the authors provide an interesting dataset the work needs linguistic revision and abbreviation - there are typos and stray punctuation marks, and the results and discussion sections would benefit from editing to improve the clarity and flow. In all, the text could be much more concise.

In accordance with ref's important comment we have considerably shortened the manuscript and generated four figs along with a report format. We have made great effort to make linguistic revision and to bring the data forward in a clearer way.

These points need to be addressed:
Several of the graphs are lacking error bars and it would be helpful to include the statistical significance on the graphs (eg Figs 3, 4, 7). Some of the bar graphs have error bars that seem too large for reliable interpretation of the data (eg Fig 3E).

In accordance with the important suggestion, all new four main figures contain statistical analysis and analyzed for statistical meaning. Several data such as old fig. 7 and old Fig. 4d-f have been omitted while old Fig 3d-f has been removed to Supp.

Figs 1-4 should be consolidated into one or 2 figures. The current volume of individual panels and

bar graphs is daunting for the reader and not proportional to the point the authors are making. As an example, couldn't the authors find a way to condense all of the individual panels in 1b into a single small bar graph?

We have extremely condensed data so as to make it more proportionate to the point we are trying to make. We have integrated the data into four main figures.

Fig 5: panels a and b don't add anything - the authors should omit them and replace with a schematic outlining the experimental plan.

These have been omitted.

Fig 7: Considering all of the caveats around interpretation of this experiment I don't think it should be included in the paper at all. In my opinion the paper would be much stronger without this data - it doesn't provide any answers and generates a lot of questions that can only be answered by speculative arguments.

In accordance with ref's suggestion these data have been omitted.

Fig 9: The message of this figure can be expressed in a single sentence. It does not add anything and should be removed.

In accordance with ref's suggestion this figure has been removed.

Referee #2 (Comments on Novelty/Model System):
The isolation of human nephron progenitors using NCAM as a marker for FACS sorting is interesting, but some of the data are of low quality and could be improved (see comments below). The fact that renal progenitor cells can differentiate into nephron cells is per se not too surprising.

Thanks for this comment. We have performed new and detailed experiments so as to improve the quality of old figs and also add new data to substantiate our results. We believe that this paper has promoted the quest for derivation of genuine nephron progenitors that can be cultured and manipulated in vitro yet retain differentiation potential.

Referee #2 (Remarks):
The present manuscript is a much improved version over the initial submission and the authors have performed added additional experiments most notably employing a second renal disease model (5/6 nephrectomy).
Listed below are still some points that should be addressed.

Response of authors to my original comment 1:
Comparative real time RT-PCR analysis of gene expression were analyzed using SDS RQ Manager 1.2 software or StepOne Software v2.1 using the well accepted method of ΔΔCT such that the expression levels of the calibrator samples, in this case SCM samples =1, therefore in each biological repeat irrespective of the absolute expression level, the calibrator is =1.

While I am not familiar with this particular software, I respectfully disagree with the authors' response. The method used allows comparison between one sample under different conditions (in this case SFM and SCM). However, I was interested in the variation between samples isolated from different kidneys. Biological samples are bound to show a certain degree of variation in gene expression and setting the 'control' sample to 1 hides this variability.

Thank you for this important comment. There is no doubt that variance exists across different human donors. We try to minimize the noise by utilizing a least three different donors for each experiment and in order to enable comparison within experiments we measure in a relative model (like fold-change one uses in microarray analysis across repeated samples). The idea in this manuscript is to show differences between two sub-populations within in the same donor/derived-culture and not between donors. It doesn't matter what donor we use, the observation is that the

NCAM1+ population is different and better than NCAM1- irrespective with absolute values (for example gene-copy number) and we calculate the average difference retrieved from each experiment to show statistical significance. Therefore, similar to fold-change, one of the samples carries a calibrator of one and the other sample describes the relative change in comparison.

Figure 2:
Images for immunostainings in Figure 2 C and D are of too low resolution. High power views should to be provided.
The staining for SIX2 is worrisome. SIX2 is a transcription factor and should be almost exclusively within the nucleus. The images and video provided does not give me this impression. Either the staining has not worked probably (what do control kidney samples look like?), or the authors only detect background staining. Incidentally, the video (confocal Z-stack) does not provide important information and should be removed.

Thanks for this important comment. We have performed new experiments on cultured mouse embryonic kidneys and on cultured human NCAM1+ cells (mouse-New Fig. 1a, human-New Fig. 2e, f). Importantly, we have included control high Six2 expressing Wilms' tumor cells and human adult kidney cells deficient in Six2 expression (New Supp. Fig. 4).
New IF experiments show that human NCAM1+ nephron progenitors are not entirely Six2+ but clearly contain the Six2+ population while showing reduced expression of E-cad compared to NCAM1- counterparts (New fig. 2). Moreover to get a detailed look of spatial relationships between Ncam1, Six2 and E-cad we have studied expression in cultured mouse embryonic kidneys. These data clearly show an overlap between Six2 and Ncam in the cap mesenchyme cells and that Ncam1 expresses beyond the Six2 domain in early nephron figures (Fig. 1a). Indeed, cultured human NCAM1+ cells contain also an NCAM1+SIX2- cell population (new Fig. 2) altogether indicating that $NCAM1^{+}$ cells likely was heterogeneous, comprising a mix of stem and more committed progenitor cells.

Figure6:

The immunostaining data for DBA, LTA and Tamm-horsfall are appreciated, although I would have preferred to see these data in co-staining with a human-specific antigen to ensure that these cells are indeed derived from the transplanted nephron progenitors.

Thank you for the important comment. Indeed optimal setting would show co-staining, nevertheless we show in previous papers coming out of the lab (Noiman, Buzhor, Organogenesis, 2011) on the CAM-renal system and in this paper human non-chick cross reactive specific marking with Ki-67 Ab (pls see new Supp. Fig. 6 f-h).

The new experiments on the 5/6Nx chronic renal disease mouse model are highly appreciated and show that human renal progenitor cells can integrate into the diseased kidney and form epithelial structures. Unfortunately, images 8j-l are difficult to interpret and it is not clear whether cells are truly integrating into damaged tubules or whether they form independent tubules that are not hooked up to the general collecting system. Higher power images using costaining of HLA and E-cadherin or HLA and tubule segment specific markers would be helpful.

Thanks for this important comment. In accordance with the referee's comment we have performed new experiments tracing outcome of hNPCs and show segment specific marker staining along with epithelial pan-cytokeratin and HLA marking. We have added segment-specific markers (New Fig. 4l, m). The EMA distal marker was also found to be a human-specific marker (New fig. 4 and new Supp. Fig. 7). This has been done on consecutive sections rather than co-staining. Nevertheless, we believe that human origin of cells is beyond doubt. We describe three forms of human cell engraftment: 1. Engraftment between tubules; 2. Engraftment into existing tubules. 3. Tubular regeneration. It is beyond this scope of this paper to determine whether they are hooked to the collecting system. However, we correlate these forms of engraftment and the production of renal epithelium with beneficial functional therapeutic outcome in when diseased mice are treated with hNPCs. According with your suggestion we have added to old images 8j-i additional images in high magnification with panCK staining (Fig. 4h, i).

Referee #3 (Comments on Novelty/Model System):

I believe there continue to be many problems in the current manuscript. There are many RT-PCR data but insufficient immunocytochemistry to confirm protein expression by the cells. The use of saline controls rather than NCAM- controls in the animal studies remains a significant problem. I have a number of additional concerns.

Thanks for these important comments. We have performed new and detailed experiments so as to supplement RQ PCR data with immunostaining. We have performed new immunostaining experiments on cultured mouse embryonic kidneys, on SFM cultures and on cultured human NCAM1+ cells (New Fig. 1a, Fig. 2, Supp Fig. 3, Supp Fig. 4). Importantly, we have included control high Six2 expressing Wilms' tumor cells and human adult kidney cells deficient in Six2 expression (New Supp Fig. 4).
New IF experiments show that human NCAM1+ nephron progenitors are not entirely Six2+ but clearly contain the Six2+ population while showing reduced expression of E-cad compared to NCAM1- counterparts (New Fig. 2). Moreover to get a detailed look of spatial relationships between Ncam1, Six2 and E-cad we have studied expression in cultured mouse embryonic kidneys. These data clearly show an overlap between Six2 and Ncam1 in the cap mesenchyme cells and that Ncam1 expresses beyond the Six2 domain in early nephron figures (New Fig. 1a). Indeed, cultured human NCAM1+ cells contain also an NCAM1+Six2- cell population altogether indicating that $NCAM1^{+}$ cells likely was heterogeneous, comprising a mix of stem and more committed progenitor cells.
In accordance with ref's suggestion for more immunostaining analysis we have performed new experiments showing in addition to FACS, staining of CD24 and EpCAM in the hFK SFM cell culture (New Supp Fig. 3).
In accordance with ref's suggestions we have now analysed by additional immunostaining the types of renal structures generated in diseased kidneys and present a more detailed analysis (New Fig. 4). Importantly, the cell therapy experiments we employed with the human nephron stem/progenitors delineated by the NCAM1 marker are in no way a measure of their stem/progenitor character. We extensively show in data and figures leading to the cell therapy part in the 5/6 Nx model that NCAM1+ cells harbor stem/progenitor and clonogenic characteristics. Only after we show that they, NCAM1+ cells, indeed harbor stem/progenitor properties when compared to their negative counterparts (including in the chick embryo CAM differentiation assay) we go on to employ these hNPCs in a stringent animal model to show that they halt disease progression. We wanted to show that the hNPCs have a functional therapeutic effect and determine whether these cells would have a beneficial effect. Once we noticed they induce a therapeutic effect we analyzed whether they indeed engraft and produce kidney epithelium. For a comparison with human CNS-SCs in a murine CNC disease model (pls see Sci Transl Med 10 October 2012:
Vol. 4, Issue 155). In FDA pre-clinical studies on human cell therapy there is no request to show that the stem/progenitor cell of interest has more effects than another cell type. The request is only a comparison to placebo - saline - as we employed in our study. I therefore, kindly approach you to be considerate in regard of this recommendation.

Referee #3 (Remarks):

The authors have responded adequately to some of my concerns. However there remain a number of issues:

1. Page 5: cells are stated to have doubling time of 32.95 hr. I find it difficult to see numbers like this with no statistical variation presented and with 2 decimal points. This is not the only place this level of precision is implied which is not realistic biologically.

Thanks for this comment. These data have been omitted.

2. Page 5: it is implied that growth in serum results in EMT because there is more fibroblast outgrowth by 7 days. This might rather just as well be related to selection of the cells from the very beginning of the isolation from human fetal kidney.

Thanks for this important comment. We have clarified accordingly (New page 6).

3. The data on Figure 1c indicate very high expression patterns of proteins. It would be much more

reassuring to see immunocytochemistry to demonstrate these staining patterns.

Thanks for this comment. We have added IHC to supplement RQ-data (Fig 1c has is now included in Supp Fig. 3 where we added additional related IF experiments). The new NCAM1 IF staining was added to new Fig. 1e.

4. Supplemental Figure 2 is not well explained in the legend nor in the text. The figure is also difficult to read because the legends are not printed at a resolution that would be necessary.

Thanks for this comment. We have re-written the manuscript so as to make more accessible and revised accordingly.

5. Figure 6. Is there any staining with any of the antibodies or lectins after NCAM- cell injections? The text on page 11 is not clear on this and there are no data presented.

Thanks for this comment. Pls note lack of kidney structure formation on the CAM with NCAM1- cells and hence no staining was performed. The data is described in New Fig. 3 and Supp Fig. 6.

6. Figures 7a and d should include error bars.

7. Why was human GAPDH used for demonstration of engraftment in the glycerol experiments and b2-microglobulin in the chronic kidney disease experiments? The authors should demonstrate human GAPDH in the chronic experiments. Furthermore, how many animals are reflected in the data presented in figure 8e?

In accordance to ref 1 suggestion and encouragement to submit in a more concise report format the acute injury model has been omitted. Cell tracking of human cells by human-specific RNA analysis (old Fig. 8e, New Fig. 5b) is a representative example of at least three experiments. We show in new Supp Fig. 7c a representative amplification plot of the same sample so as to enable a more quantitative impression of the data.

8. The authors have chosen selective measures of efficacy in the animal studies. For example there is a statistical difference in the number of animals who suffered a >15% reduction in creatinine clearance if the NCAM+ cells are injected. This reviewer would like to see the time course of creatinine clearance values for each group as I suspect the differences might be small. Why was 15% chosen? This is not standard.

Thanks for this comment. We in fact wanted to analyze what is the relative number of mice that succumb to poor outcome. Poor outcome included mortality (not-surgical related) and decreased renal function with time in treated vs. untreated mice. The statistics (Fisher test measuring relative proportion beyond chance) are in favor of the treated group when judging according to >10%, >15% and >25% GFR reduction. We show >15% as an example.

More importantly, are plots measuring the absolute change in GFR (ml/min) in each mouse compared to baseline GFR which is varying among mice. This would be the most realistic way to analyze for instance humans with CKD (with varying creatinine levels) in a clinical trial analyzing renal function after giving a certain therapeutic (for instance NEJM, N Engl J Med 2011;365:327-36). Summation of each delta GFR with time clearly unequivocally shows that treated mice show a delta GFR which is significantly favorable following the hNPC injection protocol in diseased murine hosts!

9. Figure 8h-o: How often quantitatively can the authors conclude that the hNPCs integrate into existing tubules and form new tubules by human specific marker staining? Is it not possible that some of the isolated cell staining may be related to cell death of the injected cells and uptake by other cells of the labeled debris? Also what is seen when NCAM- cells are injected? It is not appropriate to use saline as a control for these experiments without also testing NCAM- cells.

Thank you for this comment. Unfortunately there are no truly good way to quantify integration and regeneration of new human tubules in the 3D mouse kidney. We want out of our way to be sure that we indeed utilize non-cross reactive human specific marker so as to show the production of human epithelium by hNPCs in the chimeric mouse kidney. The generation of human kidney epithelium is linked to a beneficial therapeutic effect we observe with hNPCs. Clearly multiple mechanisms

beyond the generation of human kidney epithelium are likely to be responsible for the effects (anti-fibrotic??). These will be analyzed in future studies.

10. The abstract conclusion that these findings have "major potential for cell-based therapeutic strategies" ignores the potential immunological difficulties and the overall difficult ethical issues with deriving cells from human fetuses.

Thank you for this important comment. As stated in the discussion hNPCs may represent allogeneic cell therapy. This form of cell therapy harbors immunological hurdles. We have added a sentence to the discussion stating that from a clinical perspective it might be useful to initiate allogeneic cell therapy in patients already on immunosuppressive medication. Importantly, our ability to culture and expand hNPCs (possibly bank in the future) rather than utilize direct fresh cells reduce ethical considerations as fetal material will not be directly required for a specific patient. Examples from the CNS world include cultured human CNS-stem cells that are actually cultured human fetal brain CD133+ neural stem/progenitors (Uchida, Weissman, Gage). These cells have been tested in pre-clinical and clinical indications by well established groups, pls see very recent publications: Sci Transl Med 10 October 2012 4:155ra136 and Sci Transl Med. 2012 Oct 10;4(155):155ra137, respectively.

We have made a considerable effort to address the majority of the refs' suggestions and emphasize the uniqueness and novelty of our proof-of-concept experiments with human cells for translational purposes.
I hope you will now find the manuscript suitable for publication in EMBO Molecular Medicine.

3rd Editorial Decision 08 July 2013

Thank you for the submission of your revised manuscript to EMBO Molecular Medicine and I am very sorry for the long delay in getting back to you. We have now finally received the enclosed reports from the referees that were asked to re-assess it and have further discussed it within our editorial team.

As you will see Referees #1 and #2 are now supportive but Referee #3 is still not fully satisfied. We would like you to rephrase and moderate your claims as highlighted by this Referee, namely regarding points 2, 3, 4, 9. Importantly, we would appreciate if you could remove all ambiguities within the text as NCAM1+ cells is a mixed population but not true nephron progenitors. Moreover, while we find the therapeutic beneficial effect of the population upon injection in mouse models of renal disease well documented and convincing, the extent of the integration into functional nephrons remains unclear and this should also be discussed appropriately. Please also add the quantitations and controls as requested by this referee.

Please submit your revised manuscript as soon as possible together with a point-by-point response to Referee #3's comments.

NB: In order to move forward as fast as possible after receiving your revised manuscript, we would kindly ask you to make sure that:
- all figures are publication-ready (high resolution, labels readable),
- an accession number for the microarray data from GEO or ArrayExpress has been added in the Mat & Met section,
- all figures in the figure legend have a caption followed by description of the figure
- the title and authors are added to the 1st page of the Supplementary Information pdf file

In addition, please do let us know whether you would object to the publication of the point-by-point responses to referees to be published online (see below).

I look forward to reading a new revised version of your manuscript as soon as possible.

***** Reviewer's comments *****

Referee #1 (Remarks):

The manuscript is greatly improved and makes some valuable observations that will be of interest in the field. One change that needs to be made is Fig 4c - the "poor outcome" graph should be replaced with the mortality curve.

Referee #2 (Comments on Novelty/Model System):

The authors have further improved their manuscript and while I think that the therapeutic potential for these cells is still questionable and would require much more thorough experiments, I now support publication.

Referee #2 (Remarks):

The authors have largely satisfied my requests for revisions.

Referee #3 (Remarks):

The authors of the paper have reduced the number of pictures in the body of the paper to four and include seven figures and one table as supplemental data. There continue to be concerns that I have regarding the manuscript.

1. As was pointed out by one of the other reviewers, Six2 staining should be nuclear, and yet, in figure 1A, the pattern does not appear to be nuclear.
2. The authors state in the first heading of the results section that NCAM1+ cells are "highly enriched" for embryonic renal stem cell markers, however the currently presented data do not confirm this. RT-PCR data in Figure 1C and 2A shows at best low level upregulation of genes such as SIX2, OSR1, PAX2, SALL1, and WT1.
3. The authors refer to a new figure 2, where there is a very different stain pattern than there is in figure 1, which is done in the mouse. Figure 2D, the number of NCAM positive cells that are Six2 positive is very small. In figure 2E, there does appear to be some costaining between Six2 and NCAM1
4. In figure 2A, the authors state that there is a significant elevation of the nephron progenitor markers in NCAM1+ cells vs NCAM1- cells. This appears to only be true for SIX2 and WT1. The expression of OSR1 and PAX2, which are two other highly expressed markers in the nephron progenitor population, are essentially similar in NCAM1+ and NCAM1- cells. These calls into question the identity of the NCAM1+ cells as the true nephron progenitor population.
5. Comparisons between NCAM1+ and NCAM1- cell immunostaining results (Figure 2B-D) would be stronger and more believable if the authors presented quantitative data of the immunostaining.
6. One observation that is present in some of the figure panels (e.g. 2G, 2I) is that the error bar caps are not aligned with the error bar.
7. Figure 2I: in this experiment, the investigators treated cells with the NCAM1 antibody with and without DM1 toxin. The conclusion is that the reduced clonogenic capacity was related to the depletion of NCAM1 positive cells. However, there is no toxin-alone control, and it may very well be that other cells are deleted in this preparation, whether or not they express NCAM or not.
8. On page 11, the authors refer to supplemental figure 6A as containing data relating to the 5/6 nephrectomy experiments. However, it is supplemental figure 7 that contains these data.
9. The experiment performed in the mice with 5/6 nephrectomy have results presented in figure 4C. By 6 weeks after nephrectomy there is a difference in "mice with poor outcome". The determination of "poor outcome" is somewhat arbitrary however. These results also are somewhat difficult to put in context because we have no uninjected controls as a means to compare. With the small level of integration, it is not clear to what the authors attribute this improvement in function if there is one. Figure 4D shows an error bar which is larger than the measurement, which calls into question the statistical significance of these data.

3rd Revision - authors' response 17 July 2013

We have revised our manuscript in accordance with Referee #3 comments and much hope that the manuscript will be now suitable for publication in EMBO Mol Med.

In general, we have rephrased and toned down claims specifically indicating NCAM1+ cells as mixed population comprised of stem cells and more committed progenitors. This highlighted and discussed throughout the text. Importantly, we have added new experiments showing that Cited1, a nephron progenitor marker, is also significantly enriched in NCAM1+ cells (new Fig. 1). In addition, we have added better images of SIX2 staining and quantified the immunostaining signal using image software (new Fig. 2). Moreover, as the extent of the integration into functional nephrons remains unclear we discuss the need for further work to delineate differentiation-dependent vs differentiation-independent mechanism for the functional benefit we see in mice with CKD.

Referee #3 (Remarks):

The authors of the paper have reduced the number of pictures in the body of the paper to four and include seven figures and one table as supplemental data. There continue to be concerns that I have regarding the manuscript.

1. As was pointed out by one of the other reviewers, Six2 staining should be nuclear, and yet, in figure 1A, the pattern does not appear to be nuclear.

We are slightly confused by this comment as in my opinion (and apparently also of the reviewer who originally made this comment, reviewer 3 him/herself originally did not comment on this) the signal as shown is nuclear. Since this experiment already used 3 channels for the different antibodies, we could not use a nuclear counterstain in this sample. However, the Six2 signal clearly shows the nucleoli that are characteristic of mouse nuclei. To emphasize this further we have added a fifth panel to figure 1a with an enlargement showing only the Ncam1 and Six2 signals for clarity (as the fluorescent signal of the sample has already faded we have done this enlargement digitally). Personally I feel the nuclear localization of the Six2 signal is clear in the old, 4 panel version of this figure with the 5th panel being a slight overkill, but I will leave it to the editor's discretion which version would be used.

2. The authors state in the first heading of the results section that NCAM1+ cells are "highly enriched" for embryonic renal stem cell markers, however the currently presented data do not confirm this. RT-PCR data in Figure 1C and 2A shows at best low level upregulation of genes such as SIX2, OSR1, PAX2, SALL1, and WT1.

We agree that 'highly enriched' is a subjective term, and this has been removed from the header. As for the 'at best low level' up regulation of these genes, it is obvious that in isolation each gene is not sufficient for claim any enrichment for embryonic renal stem cell marker. However, enrichment for SIX2 is a clear indication of an embryonic renal stem cell character, which is further supported by enrichment for more widely expressed genes as WT1 and SALL1. We do not claim these culture conditions select for a pure stem cell population, but we maintain that these markers show enrichment for this (figure 1c). In the comparison between NCAM1+ and NCAM1- cells (figure 2a) we see indeed a much stronger enrichment. For instance, SIX2 and WT1 enrichment over, PAX2. Importantly and I am not at all sure the referee is aware of that, <u>Pax2 is not only expressed in the nephrogenic lineage but also in the ureteric bud.</u> NCAM1 which is maintained in the nephron lineage is <u>not</u> expressed in the UB lineage and therefore the fact that NCAM1+ cells do not enrich as much for the PAX2 gene (less than for as SIX2/WT1 which are not expressed in the UB domain) further validates the isolation of nephron lineage progenitors! A clarification addressing PAX2 expression in the UB has been added in results end of page 8. The same goes for OSR1 which is a <u>very early mesenchymal marker</u> that comes on in the intermediate mesoderm (from which

metanephros and originates from) and is also overexpressed in mesenchymal stem cells derived from bone marrow (Hum Mol Genet. 2011;20(21):4167-74;). N CAM11+ cells are cultured in SFM which precludes uninduced MM preventing significant OSR1 enrichment. The all idea is not to isolate early mesenchyme and MSCs but rather early epithelial nephron progenitors that would generate epithelium. In fact, in accordance with ref#3 comment we have added new experiments analyzing Cited1 expression, an additional nephron progenitor marker, in NCAM1+ cells and show it to be significantly expressed. These new data were added to Fig. 2A. Based on this we feel our claims are valid.

3. The authors refer to a new figure 2, where there is a very different stain pattern than there is in figure 1, which is done in the mouse. Figure 2D, the number of NCAM positive cells that are Six2 positive is very small. In figure 2E, there does appear to be some costaining between Six2 and NCAM1.

It is difficult to directly compare NCAM1/SIX2 co-staining from the mouse data figure 1a and the data from these isolated and disaggregated human embryonic kidney cells. As NCAM1 is expressed in several post-MET stages of nephron formation, the ratio of NCAM+SIX2- cells will increase as kidneys are older. The mouse data places the antibody signals in their spatial contexts, making it very easy to identify the NCAM1+/SIX2+ double positive cells through their localization in the cap. The important message of these data in figure 2 is that there are NCAM1+/SIX2+ cells and NCAM1+/SIX2- cells (pre-MET vs post-MET). This has been clarified in the text. In addition, according to ref#3 comments we have replaced old Fig.2D with a new Fig. 2D which shows conofocal images of SIX2 staining in NCAM11+ sorted cells and more clearly delineates localization. We believe that new Fig. 2D, the new addition of quantification of the SIX2 signal in Fig. 2E and Supp Fig 4 with appropriate controls, wilms tumor cells and adult kidney cells support our claims.

4. In figure 2A, the authors state that there is a significant elevation of the nephron progenitor markers in NCAM1+ cells vs NCAM1- cells. This appears to only be true for SIX2 and WT1. The expression of OSR1 and PAX2, which are two other highly expressed markers in the nephron progenitor population, are essentially similar in NCAM1+ and NCAM1- cells. These calls into question the identity of the NCAM1+ cells as the true nephron progenitor population.

As discussed above, SIX2, and to a lesser degree WT1, are more relevant than PAX2 and OSR1. Although PAX2 is indeed expressed in the progenitors, it is expressed in many other cell types as well, even being highly expressed in the ureteric bud. Therefore we would expect PAX2 to be less prominent, as our data indeed shows. OSR1 is expressed in the stages before SIX2 is activated, as well as in podocytes progenitors at lower levels. We would not want to be drawn into speculation whether or not OSR1 'should' be higher expressed or not (pls note that we use three different biological samples with variance across human fetal kidney samples resulting in large Y error bars as observed for OSR1, precluding statistical significance). Importantly, in accordance with ref#3 comment we have added new experiments analyzing Cited1 expression, an additional nephron progenitor marker, in NCAM1+ cells and show it to be significantly expressed! These new data were added to Fig. 2A. As argued above, the clear enrichment for SIX2 is the best possible indication for enrichment of the stem cells in the NCAM1+ population, and this is consistent with the strong enrichment for WT1 (whose expression is highly enriched in this stage). This is further clarified in the text.

5. Comparisons between NCAM1+ and NCAM1- cell immunostaining results (Figure 2B-D) would be stronger and more believable if the authors presented quantitative data of the immunostaining.

Thanks for this comment. We have used imageJ software and have added quantification data for immunostaining especially SIX2 (shown in Fig. 2E).

6. One observation that is present in some of the figure panels (e.g. 2G, 2I) is that the error bar caps are not aligned with the error bar.

We assume the misalignment was the result of moving the figure between programs and is now corrected.

7. Figure 2I: in this experiment, the investigators treated cells with the NCAM1 antibody with and without DM1 toxin. The conclusion is that the reduced clonogenic capacity was related to the depletion of NCAM1 positive cells. However, there is no toxin-alone control, and it may very well be that other cells are deleted in this preparation, whether or not they express NCAM or not.

Importantly FACS analysis shows a significant reduction in NCAM1 levels in ADC-treated cultures with abolished clonogenic function (See Supp Fig. 5).

8. On page 11, the authors refer to supplemental figure 6A as containing data relating to the 5/6 nephrectomy experiments. However, it is supplemental figure 7 that contains these data.

This is corrected.

9. The experiment performed in the mice with 5/6 nephrectomy have results presented in figure 4C. By 6 weeks after nephrectomy there is a difference in "mice with poor outcome". The determination of "poor outcome" is somewhat arbitrary however.

We have taken the phrasing of poor outcome and address the proportion of mice with reduction in creatinine clearance.

These results also are somewhat difficult to put in context because we have no uninjected controls as a means to compare.

We have used saline-treated mice as controls.

With the small level of integration, it is not clear to what the authors attribute this improvement in function if there is one. Figure 4D shows an error bar which is larger than the measurement, which calls into question the statistical significance of these data.

The significance of this data ($p<0.01$ as indicated in the figure legend) is determined by the actual measurements rather than the error bars and expectations.
Importantly, we have added the phrasing of "to a certain extent" to end of results section on Page 13 and we have also indicated in discussion that taken the level of engraftment further work needs to be performed to delineate differentiation-dependent vs differentiation-independent mechanism for the functional benefit we see in mice with CKD.

I hope you will now find the manuscript suitable for publication in EMBO Molecular Medicine.

4th Editorial Decision 19 July 2013

Thank you for the submission of your revised manuscript to EMBO Molecular Medicine and I am very sorry for the long delay in getting back to you. We have now finally received the enclosed reports from the referees that were asked to re-assess it and have further discussed it within our editorial team.

As you will see Referees #1 and #2 are now supportive but Referee #3 is still not fully satisfied. We would like you to rephrase and moderate your claims as highlighted by this Referee, namely regarding points 2, 3, 4, 9. Importantly, we would appreciate if you could remove all ambiguities within the text as NCAM1+ cells is a mixed population but not true nephron progenitors. Moreover, while we find the therapeutic beneficial effect of the population upon injection in mouse models of renal disease well documented and convincing, the extent of the integration into functional nephrons remains unclear and this should also be discussed appropriately. Please also add the quantitations and controls as requested by this referee.

Please submit your revised manuscript as soon as possible together with a point-by-point response to Referee #3's comments.

NB: In order to move forward as fast as possible after receiving your revised manuscript, we would kindly ask you to make sure that:

- all figures are publication-ready (high resolution, labels readable),
- an accession number for the microarray data from GEO or ArrayExpress has been added in the Mat & Met section,
- all figures in the figure legend have a caption followed by description of the figure
- the title and authors are added to the 1st page of the Supplementary Information pdf file

In addition, please do let us know whether you would object to the publication of the point-by-point responses to referees to be published online (see below).

I look forward to reading a new revised version of your manuscript as soon as possible.

***** Reviewer's comments *****

Referee #1 (Remarks):

The manuscript is greatly improved and makes some valuable observations that will be of interest in the field. One change that needs to be made is Fig 4c - the "poor outcome" graph should be replaced with the mortality curve.

Referee #2 (Comments on Novelty/Model System):

The authors have further improved their manuscript and while I think that the therapeutic potential for these cells is still questionable and would require much more thorough experiments, I now support publication.

Referee #2 (Remarks):

The authors have largely satisfied my requests for revisions.

Referee #3 (Remarks):

The authors of the paper have reduced the number of pictures in the body of the paper to four and include seven figures and one table as supplemental data. There continue to be concerns that I have regarding the manuscript.

1. As was pointed out by one of the other reviewers, Six2 staining should be nuclear, and yet, in figure 1A, the pattern does not appear to be nuclear.
2. The authors state in the first heading of the results section that NCAM1+ cells are "highly enriched" for embryonic renal stem cell markers, however the currently presented data do not confirm this. RT-PCR data in Figure 1C and 2A shows at best low level upregulation of genes such as SIX2, OSR1, PAX2, SALL1, and WT1.
3. The authors refer to a new figure 2, where there is a very different stain pattern than there is in figure 1, which is done in the mouse. Figure 2D, the number of NCAM positive cells that are Six2 positive is very small. In figure 2E, there does appear to be some costaining between Six2 and NCAM1
4. In figure 2A, the authors state that there is a significant elevation of the nephron progenitor markers in NCAM1+ cells vs NCAM1- cells. This appears to only be true for SIX2 and WT1. The expression of OSR1 and PAX2, which are two other highly expressed markers in the nephron progenitor population, are essentially similar in NCAM1+ and NCAM1- cells. These calls into question the identity of the NCAM1+ cells as the true nephron progenitor population.
5. Comparisons between NCAM1+ and NCAM1- cell immunostaining results (Figure 2B-D) would be stronger and more believable if the authors presented quantitative data of the immunostaining.

6. One observation that is present in some of the figure panels (e.g. 2G, 2I) is that the error bar caps are not aligned with the error bar.
7. Figure 2I: in this experiment, the investigators treated cells with the NCAM1 antibody with and without DM1 toxin. The conclusion is that the reduced clonogenic capacity was related to the depletion of NCAM1 positive cells. However, there is no toxin-alone control, and it may very well be that other cells are deleted in this preparation, whether or not they express NCAM or not.
8. On page 11, the authors refer to supplemental figure 6A as containing data relating to the 5/6 nephrectomy experiments. However, it is supplemental figure 7 that contains these data.
9. The experiment performed in the mice with 5/6 nephrectomy have results presented in figure 4C. By 6 weeks after nephrectomy there is a difference in "mice with poor outcome". The determination of "poor outcome" is somewhat arbitrary however. These results also are somewhat difficult to put in context because we have no uninjected controls as a means to compare. With the small level of integration, it is not clear to what the authors attribute this improvement in function if there is one. Figure 4D shows an error bar which is larger than the measurement, which calls into question the statistical significance of these data.

4th Revision - authors' response 29 July 2013

We submitted the new version of our manuscript: EMM-2012-01584----V5, According to your instructions. The microarray data was deposited to the Gene Expression Omnibus (GEO), under accession GSE49101 (we also mentioned it in the Supp file).
