# Supplementary material for: Identification of human nephron progenitors capable of generation of kidney structures and functional repair of chronic renal disease
Source: EMBO Mol Med. 2013 Sep 2;5(10):1556–68. doi: 10.1002/emmm.201201584 (PMC3799579; doi:10.1002/emmm.201201584)
Supplement: Supplementary file 2 [file emmm0005-1556-SD2.pdf]

Identification of human nephron progenitors capable of generation of kidney structures and functional repair of chronic renal disease

Running title: **Renal developmental progenitors**

Orit Harari-Steinberg[1]*, Sally Metsuyanim[1,2]*, Dorit Omer[1,3], Yehudit Gnatek[1], Oren Pleniceanu[1,3], Rotem Gershon[1,3], Sara Pri-Chen[1], Derya Deniz Ozdemir[4], Yaniv Lerenthal[5], Tzahi Noiman[2], Herzel Ben-Hur[6,7] , Zvi Vaknin[7], David F Schneider[7], Bruce J Aronow[8], Ronald S Goldstein [2], Peter Hohenstein[4], Benjamin Dekel[1,3,9]

1. The Pediatric Stem Cell Research Institute, Edmond & Lily Safra Children's Hospital, Sheba Center for Regenerative Medicine, Sheba Medical Center, Israel
2. Mina and Everard Goodman Faculty of Life Sciences, Bar-IlanUniversity, Ramat-Gan, Israel
3. Sackler School of Medicine, Tel Aviv University, Israel
4. The Roslin Institute, University of Edinburgh, Easter Bush Campus, Midlothian, EH25 9RG, United Kingdom
5. Cancer Research Center, Sheba Medical Center, Israel
6. L.E.M Laboratory of early detection, Nes Ziona Israel
7. Department of Obstet. & Gynecology, Assaf Harofeh, Israel
8. Division of Molecular and Developmental Biology, Department of Pediatrics, University of Cincinnati, Childrens Hospital Medical Center, Cincinnati, USA
9. Division of Pediatric Nephrology, Edmond& Lily Safra Children's Hospital, Sheba Medical Center, Israel

[*] Orit Harari-Steinberg and Sally Metsuyanim share equal contribution.

Correspondence:

Benjamin Dekel M.D., Ph.D.

Pediatric Stem Cell Research Institute

Edmond and Lili Safra Children's Hospital,

Sheba Medical Center,

Tel Hashomer, 52621, Israel

Tel # 03-5302445; Fax # 03-5303637

E-mail: binyamin.dekel@sheba.health.gov.il or benjamin.dekel@gmail.com

Table of content:

1. Supplementary materials and methods…………………………………………………………………………… 5

2. Supp Fig. 1 …… 8

3. Supp Fig. 2 …… 9

4. Supp Fig. 3 …… 10

5. Supp Fig. 4 …………………………………………………………………………………………………..…… 11

6. Supp Fig. 5 ……. 12

7. Supp Fig. 6 ……. 13

8. Supp Fig. 7 ……... 14

9. Supp Table 1……

10. Supp Table 2…… 16

11. Supp Table 3…… 17

12. Supp Table 4…… 18

13. Supp Table 5……. 19

Supplementary materials and methods:

(2'z,3'e)-6-Bromoindirubin-3'-oxime (BIO) treatment: NCAM1$^+$ cells sorted by magnetic beads as described below were plated on a 6-well plate (2.5X10^5 cells/well) with or without 1µg Fibronectin plate coating (Biological Industries) and cultured in SFM supplemented with 5uM BIO(Tocris Bioscience). After seven days in the culture, the number of cells was evaluated using Trypan blue.

Bone morphogenic protein 7 (BMP7) treatment: NCAM1$^+$ cells sorted by magnetic beads as described below were plated with or without 1µg Fibronectin plate coating and cultured in SFM supplemented with 50ng/ml BMP7 (Peprotech Asia). After seven days in culture, the number of cells was evaluated using Trypan blue.

Microarray analysis: Total RNA from 3 different samples of whole hFK cells cultured in SFM and NCAM1+ cells (magneticly sorted from the same hFK culture) was purified using Trizol reagent (Invitrogen) according to manufacturer's recommendations. All experiments were performed using Affymetrix HU GENE1.0st oligonucleotide arrays http://www.affymetrix.com/support/technical/datasheets/gene_1_0_st_datasheet.pdf. RNA was used to prepare biotinylated target DNA, according to manufacturer's recommendations. The target cDNA generated from each sample was processed as per manufacturer's recommendation using an AffymetrixGeneChip Instrument System https://www.affymetrix.com/support/downloads/manuals/wt_sensetarget_label_manual.pdf. The quality and amount of starting RNA was

confirmed using an agarose gel or by Bioanalyser(Agilent). After scanning, array images were visually assessed to confirm scanner alignment and the absence of significant bubbles or scratches on the chip surface. The signals derived from the array were analyzed using various quality assessment metrics. Quality control measures were performed according to manufacturer's recommendations. Gene level RMA sketch algorithm (Affymetrix Expression Console and Partek Genomics Suite 6.2) was used for crude data generation. Significantly changed genes were filtered as changed by at least 2 fold (P value 0.05). Genes were filtered and analyzed using unsupervised hierarchical cluster analysis and supervised hierarchical cluster analysis (Partek Genomics Suite and SpotfireDecisionSite for Functional Genomics; Somerville,MA) to get a primary evaluation of the data. Further processing included functional analysis and over- representation calculations based on Gene Ontology and publication data: *DAVID Bioinformatics Resources 6.7*http://apps1.niaid.nih.gov/David/upload.asp, Ingenuity, Database for Annotation (GO), Visualization, and Integrated Discovery. Over- representation calculations are done using Ease. The microarray data was deposited to the Gene Expression Omnibus (GEO), under accession GSE49101.

GUDMAP database analysis: Genomic data for the expression of Ncam1 in developing kidney compartments were retrieved from the GUDMAP database (Harding et al, 2011) using their series of 53 MOE 430, plus two laser-captured and flow-sorted developing kidney samples, as described in Brunskill et al., 2008 (Brunskill et al, 2008). This data set encompassed the following samples: e11.5_metaneph mesenche11.5_ureteric bud, e12.5_Renal vesicles, e13.5_podocyte cells, e15.5_anlage of loop of Henle, e15.5_Cap mesenchyme, e15.5_cortic collect duct, e15.5_earlyproximal tubule, e15.5_Endothelial cells, e15.5_Medullary collecting duct,e15.5_Pelvic Mesenchyme, e15.5_Peripheral

blastema, e15.5_Podocyte cells,e15.5_Proximal Tubules, e15.5_S-shaped body, e15.5_stage III -IV renal corpuscle, e15.5_ureter tip_flank cells, e15.5_cortic collecting duct cells, e15.5_Ureteral Smooth Muscle. The RMA-normalized expression table from these was then analyzed by Pearson correlation to identify other probesets and corresponding gene transcripts that were most similarly regulated to the 1426864_a_at probe for Ncam1 using a matrix of all individual samples. Geneset enrichment analysis was carried out using Toppgene (Chen et al, 2009) and biological network analysis for shared connectivities across the developing kidney Ncam1-correlated gene set was carried out using Toppcluster(Kaimal et al, 2010) .

IMGN901 dosage screening: hFK cells were cultured with SFM in a 96-well plate (6000 cells per well). After one day, a single dose of IMGN901 (1.675uM, 0.837uM, 0.418uM, 0.2uM, 0.1uM, 52nM, 26nM, 13nM, 6.5nM, 1.6nM or 0) (ImmunoGen, Inc) was added to the culturing medium. After four days in the culture, changes in cell proliferation were evaluated using MTS, as described above. Another preliminary assay tested other IMGN901 doses (55nM, 45nM, 35nM, 25nM or 0). After four days in the culture, changes in NCAM1+ cell fraction were evaluated using FACS assay, as described above.

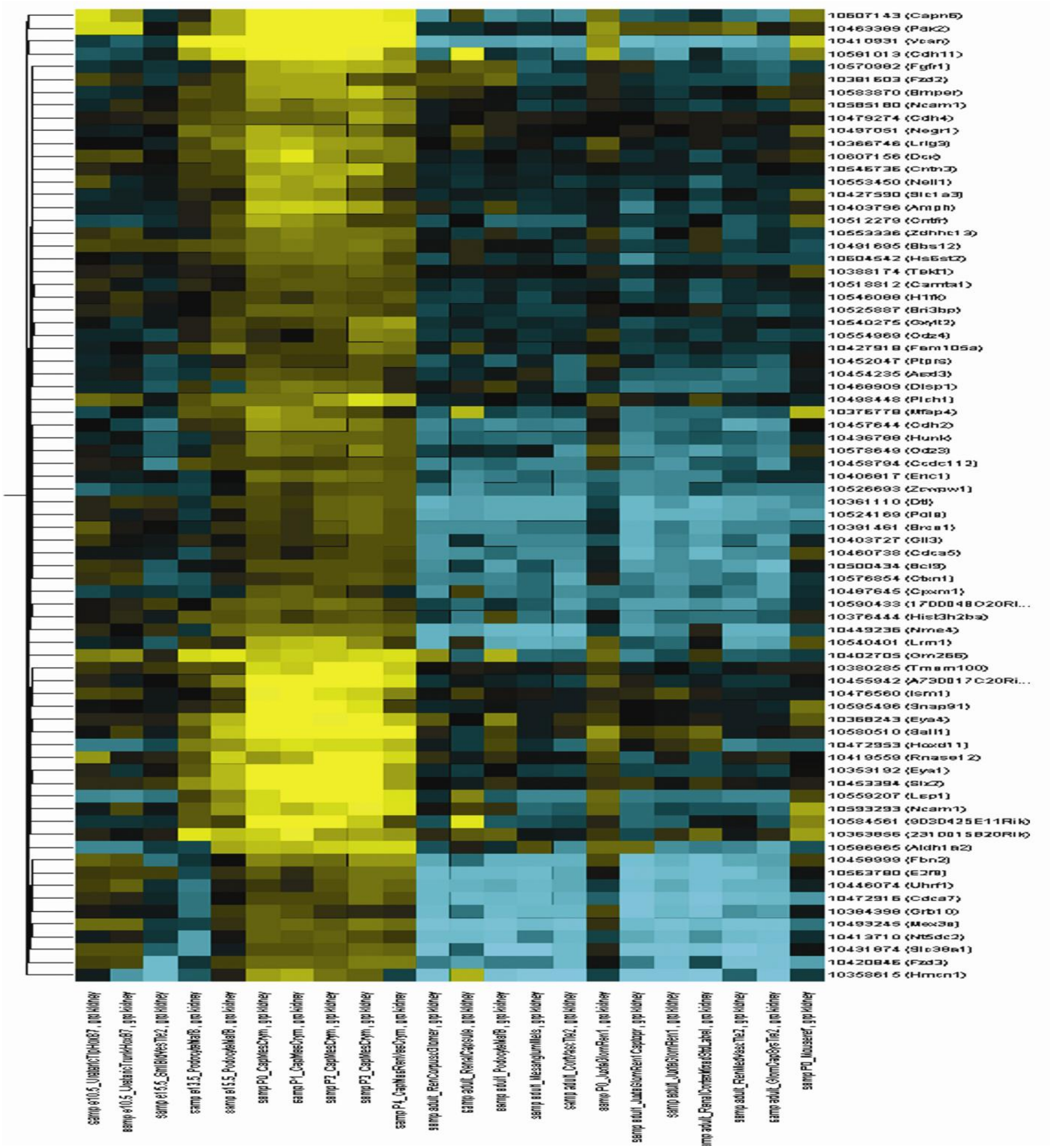

Supp Fig. 1: Gene expression coordinately regulated with NCAM1 during mouse kidney development. HeatMap of gene expression in different renal compartments and time points during mouse kidney development and adulthood, that their expression was coordinately regulated with NCAM1 expression. Genomic data was retrieved from the GUDMAP database (Harding et al, 2011) and analyzed using Pearson correlation assay across the normalized gene expression profiles.

Supp Fig. 2: NCAM1 expression module. A scheme of the NCAM1 module network. Genomic data was retrieved from GUDMAP database (Harding et al, 2011) and analyzed using Toppcluster (Kaimal et al, 2010).Genes in the Ncam1 expression module that are known to play a role in neurogenesis at some level are highlighted.

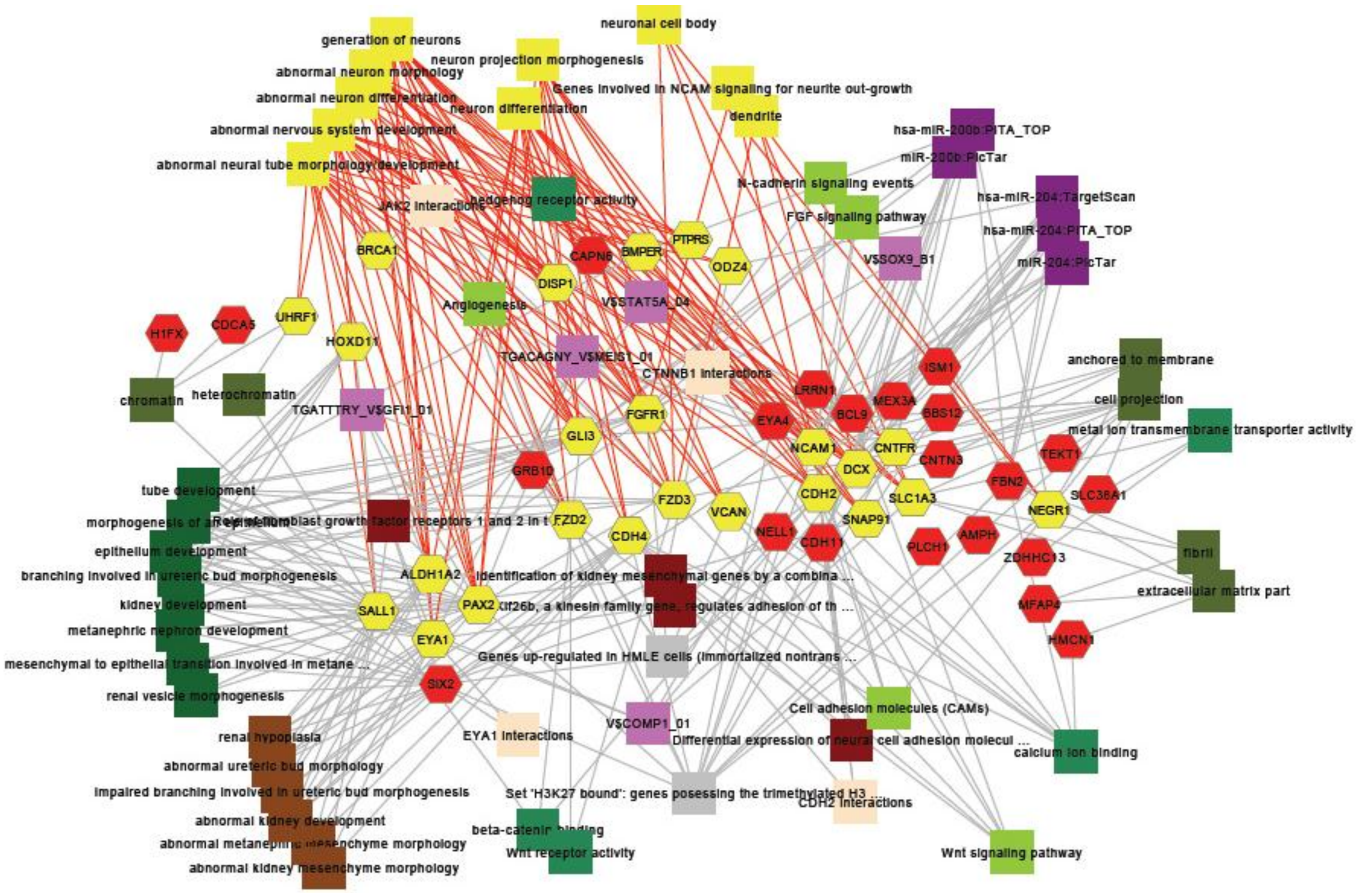

Supp Fig. 3: Characterization of hFK cells (A) FACS analysis of surface marker expression in hFK cell cultures. Each marker was tested on at least three independent samples. Data was calculated as the average percentage of expressing cells ±SDEV. **(B-C)** Immunofluorescence staining of hFK cells cultured with SFM for CD24 (B, red) and EpCAM (C, green). Nuclei were stained with Dapi (blue). Images were obtained using Olympus DP72 camera attached to Olympus BX51 fluorescence microscope and processed via cellSens standard software. **(D-E) Isolation of NCAM1+ cells from hFK cells.** Representative FACS sorting analysis of NCAM1 expression in hFK cells cultured in SFM at Passage #1. Data are presented in histogram graphs, mean fluorescence intensities (MFI) and percentage of NCAM1 expressing cells. Shown are NCAM1 (right panel) and isotype control (left panel) staining **(D)**. **(E)** Validation of sorting purity of NCAM1- (left panel) and NCAM1+ (right panel) sub-populations. **(F) Characterization of NCAM1 cells sorted from SCM cultures.** qRT-PCR analysis of gene expression in NCAM1 cell fractions grown in SCM. Normalization was performed against control *GAPDH* expression and RQ calculated relative to NCAM1$^-$ cell fraction. Data were analyzed using SDS 3.2 software and presented as average RQ±SDEV of three replicates. ***P<0.001, *P<0.05 versus NCAM1−.

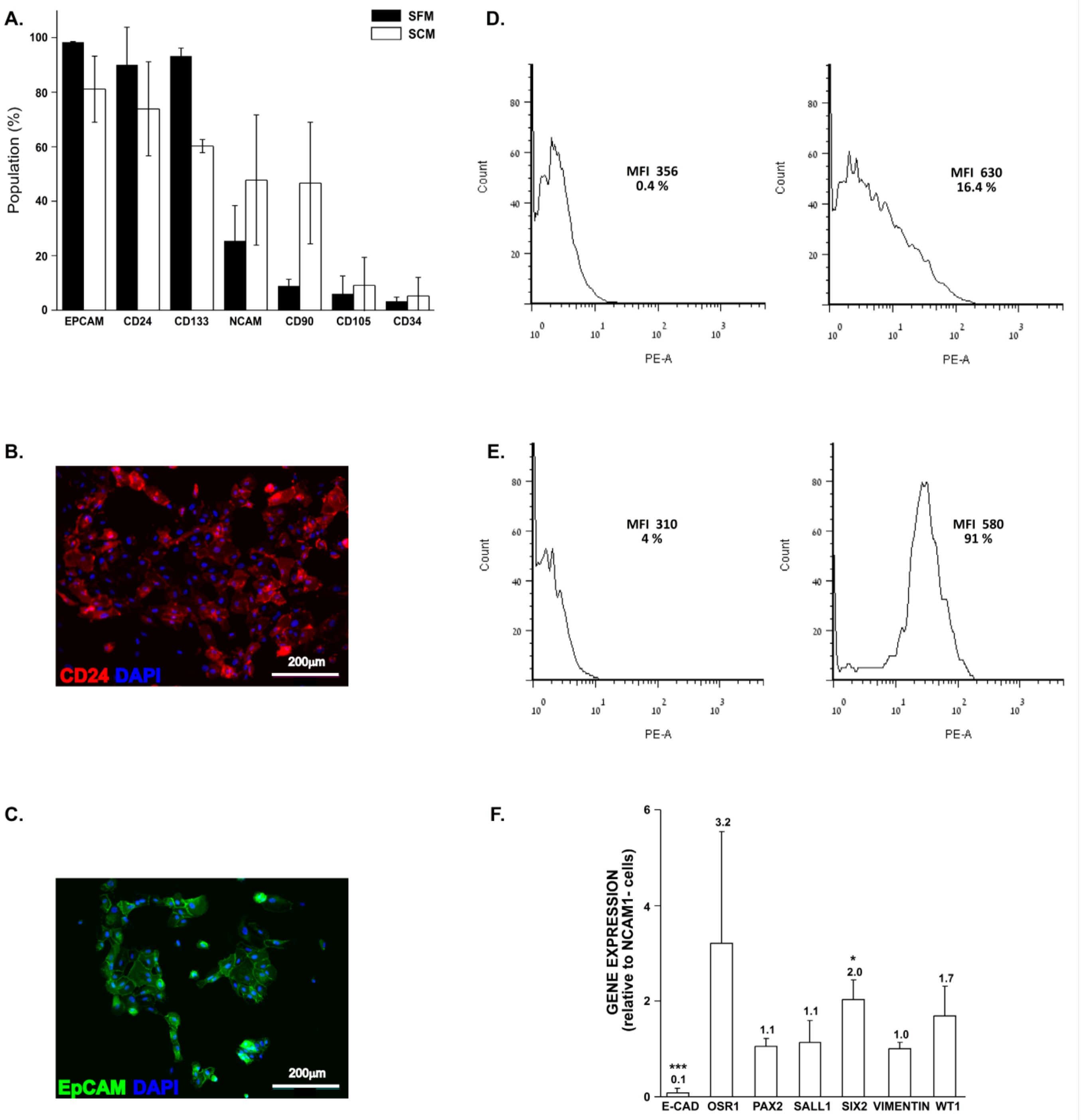

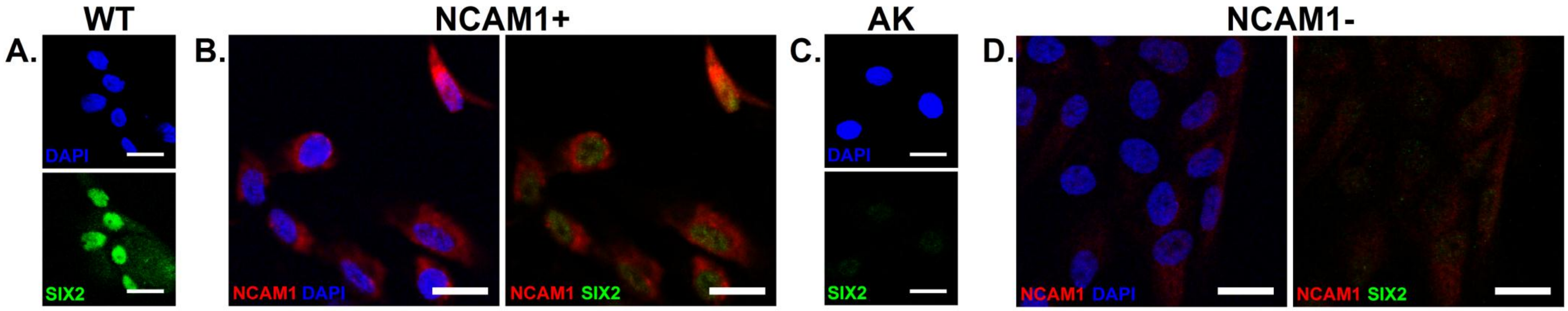

Supp Fig. 4: Calibration of immunoflorescence staining of SIX2. Double IF staining of SIX2 (green) and NCAM1 (red) in hFK NCAM1+ (B) and NCAM1- (D) sorted cells. Cells from primary cultures of wilms tumor (A) or adult kidney (D) were used as positive or negative controls (respectively) of the SIX2 staining. Nuclei were stained with Dapi (blue). Images were obtained using Zeiss SLM 510 microscope and processed in ImageJ/Fiji software. Scale: 50µm.

Supplementary Fig. 5: In vitro characterization of NCAM+ cells. **(A)** Representative graph of self-renewal in NCAM1+ cells sorted from hFK that was cultured in SCM. Data are presented for each passage as relative number of clones developed from the total number of cells plated. **(B)** Percentage of surviving NCAM1$^+$ cells after sorting and culturing with or without BMP7 for 7 days. Each treatment was tested on three independent samples. Number of cells was calculated as percentage of the control group and presented as average of three replicates ±SDEV. **(C)** Percentage of surviving NCAM1$^+$ cells after sorting and culturing with or without BIO. Number of cells was calculated as percentage of the control group. **(D)** Proliferation analysis of hFK cells treated with different dosages of IMGN901 (0-1.675uM) as demonstrated by MTS proliferation assay. OD levels are presented as percentage of the control sample (0uM). **(E)** FACS analysis of the NCAM1 population in hFK treated with different dosages of IMGN901 (0-55nM). Results are presented as percentage of the control sample (0uM). Abbreviations: FIB = fibronectin coating.

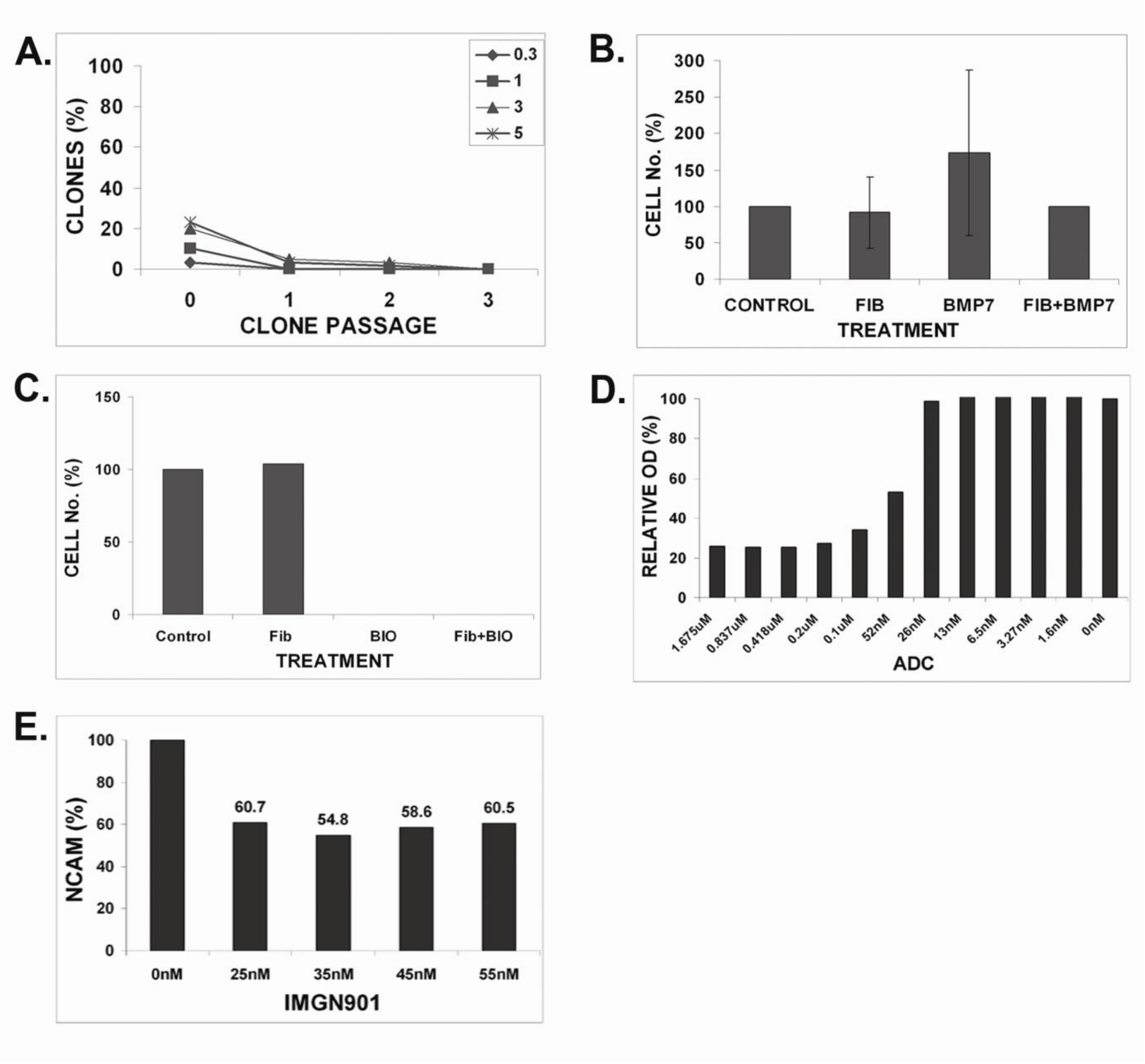

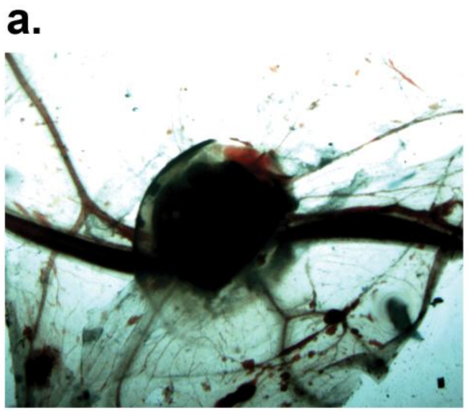

b.

Cell (X10^6)/Egg	Replicates	Visible Grafts	Grafts with tubule-like formations
10	8	8/8	0/8
5	10	10/10	1/8
2.5	5	5/5	3/5
1.25	3	3/3	0/3
0.35	3	3/3	0/3

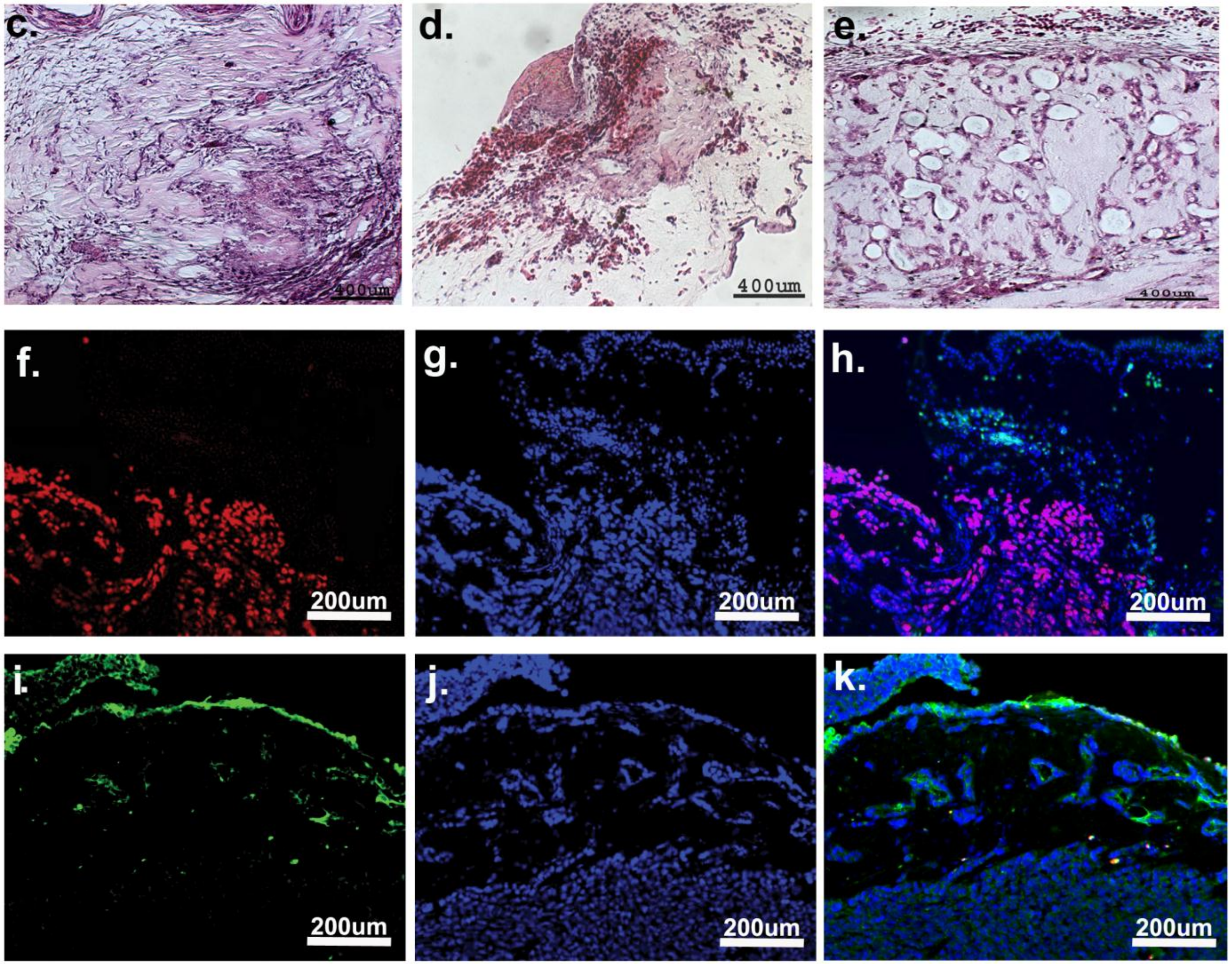

Supp Fig. 6: Renal differentiation of hFK cells on chick CAM. (A) hFK cells grafted on the chick CAM. Graft images were obtained using Scion Corporation color digital camera attached to Olympus SZX12. **(B)** Summarizing table of the amount of hFK cells grafted on the CAM (grafted cells, replicates, visible grafts and grafts with tubule-like formations). **(C-E)** H&E staining of paraffin embedded sections of 0.35X10^6 (C), 1.25X10^6 (D) and 2.5X10^6 (E) grafted cells. Images were obtained using Scion color digital cameras attached to Olympus BX51TF microscope. **(F-K)** IF staining of Ki67 (F-H, red) and pan-CK **(**I-K, green**)** in paraffin embedded sections of 2.5X10^6 cell grafts. Hoechst 33342 (Blue) was used for nuclear staining. Images were obtain using Olympus AX70 motorized microscope and spectral unmixing using multispectral imaging system (NuanceFX camera and software).

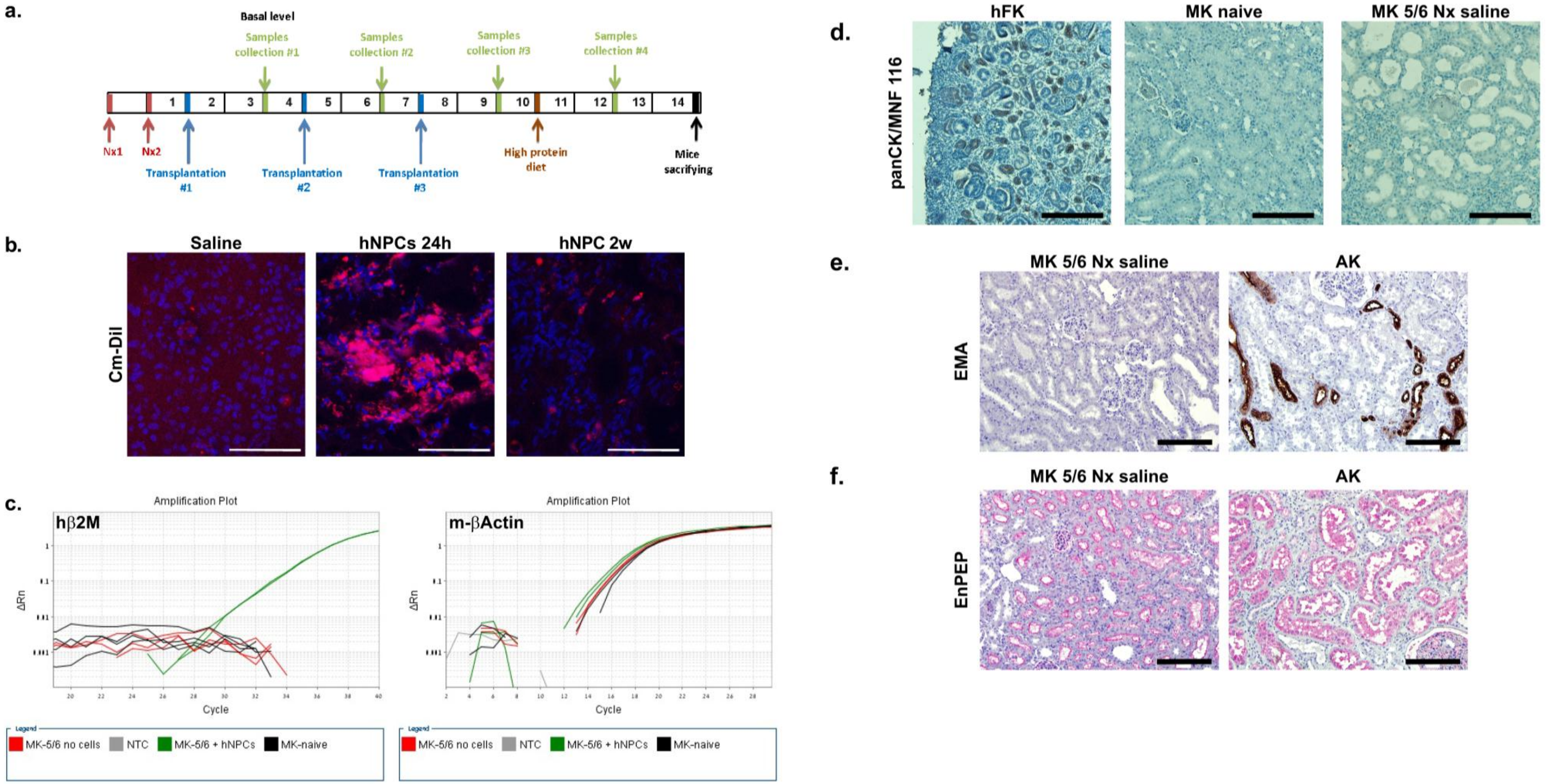

Supp Fig 7: Chronic renal disease model of 5/6-Nephrectomy (5/6Nx) in NOD/SCID mice (A) S chema of in vivo experiments:5/6Nx in NOD/SCID mice was performed in two steps (Nx1-unilateral nephrectomy & Nx2-2/3 nephrectomy). One week after the second step, hNPCs were injected directly into the remnant kidney (transplantation #1-3) in three cycles, with three weeks intervals between each injection. Blood and urine were collected two weeks after each cell injection (sample collection #1-3). Starting on the 10th week mice were fed by high protein

food followed by another blood and urine sample collection (#4). 14 week after the second step of the Nx the mice were sacrificed and the kidneys were removed for IHC or gene expression analysis. (B) hNPCs were labeled with CM-Dil Cell Tracker Mice injected with saline served as controls. Sections of the injected kidneys were then analyzed by confocal microscopy. While saline injected kidney show no detectable signal, hNPCs are seen in the injected kidneys as soon as 24h after the injection and are still detectable at 2 weeks. (C) RT-PCR for human-specific β2-microglobulin (*hB2m*) was performed to validate the presence of human cells in the injected mouse kidney. Representative Real time PCR amplification plot demonstrates amplification in the hNPC-injected mouse kidneys, but not in untreated (naïve) or saline-injected kidneys. Mouse β–Actin (*mβ–actin*) served as endogenous control. (D) IHC with human specific antibody for pan-Cytokeratin /MNF116 is shown in positive control hFK tissue (left panel), and in two negative control NOD/SCID kidney tissues: healthy/naive mouse kidney (MK-naïve, middle panel) and in 5/6 Nx kidney injected with saline (MK-5/6 saline, right panel). (E) IHC with human specific antibody for EMA in MK-5/6 saline (right panel) and in positive control human adult kidney (AK) tissue (right panel). (F) IHC with human antibody for ENPEP is shown in both MK-5/6 saline (left panel) and AK (right panel) tissues. Bar represents 200micron.

Supp Table 2: Differential expressed genes in NCAM1+ cells

Biological process	Gene	Reference
positively regulation cell cycle/cell division/cell growth	p21 protein-activated kinase 3	
positively regulation cell cycle/cell division/cell growth	CCA2	
positively regulation cell cycle/cell division/cell growth	CDC6	(Boronat & Campbell, 2008)
positively regulation cell cycle/cell division/cell growth	CDKN3	
positively regulation cell cycle/cell division/cell growth	BUB1	(Taylor et al, 2001)
positively regulation cell cycle/cell division/cell growth	CEP55	(van der Horst et al, 2009)
positively regulation cell cycle/cell division/cell growth	NUF2	(Sundin et al, 2011)
positively regulation cell cycle/cell division/cell growth	SIM1	
positively regulation cell cycle/cell division/cell growth	PDZ-binding kinase	(Park et al, 2010)
positively regulation cell cycle/cell division/cell growth	EEF1B2	(Byun et al, 2009)
positively regulation cell cycle/cell division/cell growth	MKI-67	
positively regulation cell cycle/cell division/cell growth	PBX2	(Capellini et al, 2008)
positively regulation cell cycle/cell division/cell growth	TOP2A	(Pode-Shakked et al, 2009)
positively regulation cell cycle/cell division/cell growth	ETV1	
positively regulation cell cycle/cell division/cell growth	STOML2	(Song et al, 2012
terminal epithelial differentiation	CDH1	
terminal epithelial differentiation	EpCAM	
terminal epithelial differentiation	Grhl2	

Supp Table 3: Primary antibodies used in FACS assays

**Antibody**	**Marker identified**	**Isotype control**	**Manufacturer**
CD24-PE	CD24	Mouse IgG1	eBioscience
FITC anti-human CD34	CD34	Mouse IgG2a	MiltenyiBiotec
PE anti-human CD56 (N-CAM, NCAM1)	NCAM1	Mouse IgG2a,κ	eBioscience
FITC mouse anti-human CD90	Thy-1	Mouse IgG1,κ	BD Biosciences
CD133/1 (AC133)-APC	CD133	Mouse IgG1	MiltenyiBiotec
CD326 (EpCAM)- FITC	EpCAM	Mouse IgG1	MiltenyiBiotec
FITC mouse anti-human CD105	CD105	Mouse IgG1	Serotec Inc.

Supp Table 4: Secondary antibodies used in the FACS assays

**Reagent**	**Manufacturer**
Avidin-Fluorescein (Avidin-FITC)	R&D Systems, Inc.
Streptavidin-Allophycocyanin (SA-APC)	R&D Systems, Inc.
Alexa Fluor 647 goat anti-mouse	Invitrogen
Alexa Fluor 488 goat anti-mouse	Invitrogen

Supp Table 5: Antibodies used in IHC or IF staining

Primary Antibody	Manufacturer	Secondary fluorescent Antibody	Manufacturer
Biotinylated lotus tetragonolobus lectin (lotus)	Vectorlabs	Strepavidin-Cy2	Jackson Immunoresearch
Biotinylated dolichos biflorus agglutinin (dolichos)	Vectorlabs	Strepavidin-Cy2	Jackson Immunoresearch
Sheep anti-human Tamm-horsfall glycoprotein polyclonal (THG)	Vectorlabs	-	-
SIX2 Rabbit anti-Human Polyclonal (aa264-277) Antibody	LifeSpan Biosciences	Alexa Fluor® 488 Donkey Anti-Rabbit IgG (H+L)	Life technologies
Purified Mouse Anti-E-Cadherin	BD transduction Laboratories	Alexa Fluor® 594 Goat Anti-Mouse IgG2a (γ2a)	Life technologies
Monoclonl anti-NCAM	Sigma	Alexa Fluor® 647 Goat Anti-Mouse IgG1 (γ1)	Life technologies
SIX2 Antibody	Novus	Alexa 488 conjugated anti-mouse IgG	Jackson
Anti-Vimentin antibody [RV202]	abcam	Alexa 488 conjugated anti-mouse	Jackson
E-Cadherin (24E10) Rabbit mAb	Cell Signaling	Alexa 550 conjugated or anti-rabbit IgG	Jackson

Monoclonal Mouse Anti-Human Cytokeratin	DAKO	ImmPRESS™ system: anti-mouse-HRP	Vector
Anti-MHC class I antibody [EP1395Y]	abcam	Alexa Fluor® 488 Donkey Anti-Rabbit IgG (H+L) for IF or ImmPRESS™ Anti-Rabbit Alkaline Phosphatase for IHC	Life technologies (IF) or Vector (IHC)
Monoclonal Mouse Anti-Human Cytokeratin	DAKO	ImmPRESS™ system: anti-mouse-HRP	Vector
EMA (E29)	Cell Marque	ImmPRESS™ system: anti-mouse-HRP	Vector
Anti-ENPEP antibody produced in rabbit	Sigma	ImmPRESS™ Anti-Rabbit Alkaline Phosphatase	Vector
CD56 (NCAM1) antibody		ImmPRESS™ Anti-Rabbit Alkaline Phosphatase	Vector
